# T cell-intrinsic role for Nod2 in protection against Th17-mediated uveitis

**DOI:** 10.1038/s41467-020-18961-0

**Published:** 2020-10-26

**Authors:** Ruth J. Napier, Ellen J. Lee, Michael P. Davey, Emily E. Vance, João M. Furtado, Paige E. Snow, Kimberly A. Samson, Sydney J. Lashley, Brieanna R. Brown, Reiko Horai, Mary J. Mattapallil, Biying Xu, Michelle C. Callegan, Luke S. Uebelhoer, Christina L. Lancioni, Richard K. Vehe, Bryce A. Binstadt, Justine R. Smith, Rachel R. Caspi, Holly L. Rosenzweig

**Affiliations:** 1 VA Portland Health Care System, Portland, OR 97239 USA; 2 Department of Molecular Microbiology and Immunology, Oregon Health and Science University, Portland, OR 97239 USA; 3 Department of Medicine, Oregon Health and Science University, Portland, OR 97239 USA; 4 Division of Ophthalmology, Ribeirão Preto Medical School, University of São Paulo, Butanta, Ribeirão Preto Brazil; 5 Department of Public Health, Oregon Health and Science University, Portland, OR 97239 USA; 6 Providence Cancer Institute, Portland, OR 97213 USA; 7 Providence Medical Group, Portland, OR 97213 USA; 8 Laboratory of Immunology, NEI, NIH, Bethesda, MD 20814 USA; 9 Department of Ophthalmology, University of Oklahoma Health Sciences Center, Oklahoma, OK 73104 USA; 10 Dean A. McGee Institute, Oklahoma City, OK 73104 USA; 11 Department of Pediatrics, Oregon Health and Science University, Portland, OR 97239 USA; 12 Department of Pediatrics, University of Minnesota and the University of Minnesota Masonic Children’s Hospital, Minneapolis, MN 55455 USA; 13 Center for Immunology and Department of Pediatrics, University of Minnesota, Minneapolis, MN 55455 USA; 14 College of Medicine and Public Health, Flinders University, Adelaide, SA 5042 Australia

**Keywords:** Autoimmunity, NOD-like receptors, T-helper 17 cells, Signal transduction

## Abstract

Mutations in nucleotide-binding oligomerization domain-containing protein 2 (NOD2) cause Blau syndrome, an inflammatory disorder characterized by uveitis. The antimicrobial functions of Nod2 are well-established, yet the cellular mechanisms by which dysregulated Nod2 causes uveitis remain unknown. Here, we report a non-conventional, T cell-intrinsic function for Nod2 in suppression of Th17 immunity and experimental uveitis. Reconstitution of lymphopenic hosts with Nod2^−/−^ CD4^+^ T cells or retina-specific autoreactive CD4^+^ T cells lacking Nod2 reveals a T cell-autonomous, Rip2-independent mechanism for Nod2 in uveitis. In naive animals, Nod2 operates downstream of TCR ligation to suppress activation of memory CD4^+^ T cells that associate with an autoreactive-like profile involving IL-17 and Ccr7. Interestingly, CD4^+^ T cells from two Blau syndrome patients show elevated IL-17 and increased CCR7. Our data define Nod2 as a T cell-intrinsic rheostat of Th17 immunity, and open new avenues for T cell-based therapies for Nod2-associated disorders such as Blau syndrome.

## Abstract (web summary)

How mutations in the microbial receptor NOD2 induce Blau syndrome in humans and related uveitis is unclear. Here the authors show, using Nod2-deficient mice and experimental uveitis, that Nod2 negatively regulates T cell activation and transcription of autoimmunity-related genes to suppress Th17 responses and uveitis.

## Introduction

Uveitis encompasses a group of immune-mediated diseases affecting primarily the uvea of the eye. It is an often painful condition that can result in irreversible ocular damage and permanent vision loss^1^, and constitutes a leading cause of blindness in developed countries. Therefore, gaps in our understanding of mechanisms underlying uveitis are of concern. Uveitis can occur in isolation, as in the case of the autoimmune disorder sympathetic ophthalmia; or it can also occur in systemic HLA (human leukocyte antigen)-associated arthritic conditions such as sarcoidosis, ankylosing spondylitis, psoriasis, or Behçet’s disease. A number of newly emergent monogenic autoinflammatory diseases, such as cryopyrin-associated period syndromes (CAPS), familial Mediterranean fever, and Blau syndrome^2^, are similarly characterized by inflammation of the skin, joints, and eyes in conjunction with periodic fevers. Many of the mutations associated with these conditions reside in NOD-like receptors (NLRs), which are intracellular innate receptors vital to host defense against infection and toxic substances^3^; hence, it is thought that such diseases arise from dysfunction in innate immunity.

Mutations in nucleotide-binding oligomerization domain-containing protein 2 (NOD2), one of the first NLRs to be described, have been identified as the genetic cause of Blau syndrome^4^. Uveitis in Blau syndrome manifests early in life, is granulomatous, can involve the entire uveal tract (comprised of the iris, ciliary body, choroid), as well as the retina, and is associated with visual morbidities such as cataract, glaucoma, and retinal detachment^5^. Uveitis in Blau syndrome is also accompanied by polyarticular arthritis and papular skin rashes^6,7^. To date, approximately 13 missense mutations have been identified within the central nucleotide-binding and oligomerization domain (NOD) in Blau syndrome patients. However, the details as to how NOD2-dysregulation results in Blau syndrome remain unknown. Currently, treatment includes life-long corticosteroid therapy in combination with immunosuppressive drugs (e.g., methotrexate or biologic agents), which have shown not only limited efficacy, but also high toxicity. Thus, a more thorough understanding of the immunopathogenesis of Blau syndrome is needed.

Nod2 is well-documented as a critical mediator of host defense against infection from various bacteria (e.g., *Listeria*, *Salmonella*, and *Staphylococcus*)^8^ via its ability to sense the cell wall components peptidoglycan (PGN) and its derivative N-acetyl-muramyldipeptide (MDP)^3^. Receptor-interacting protein 2 (Rip2) kinase is an adapter protein essential for Nod2-activation of signaling pathways involving NF-κB, MAPK p38, and JNK^3^. This “canonical” Nod2/Rip2-coupled signaling axis has been established in a number of cell types, including myeloid cells and intestinal epithelial cells, in which it promotes innate antimicrobial responses, adaptive T helper (Th) 17 immunity, and intestinal homeostasis^8,9^. To date, Nod2-regulation of Th17 immunity is understood to occur through antigen-presenting cells (APCs) such as dendritic cells (DCs), which are critical for T cell priming and Th17 differentiation of naïve CD4^+^ T cells^10^. In contrast, a direct function of Nod2 within T cells, where it is also expressed^11–15^, remains unresolved due to conflicting findings^12,16^. Since the connection between NOD2 and uveitis underscores an essential function for NOD2 in ocular homeostasis, we sought to delineate the cellular mechanism by which Nod2 disruption causes uveitis using experimental autoimmune uveitis (EAU), in which mice are immunized with the retinal antigen, interphotoreceptor retinoid-binding protein (IRBP)^17^.

Here, we demonstrate a role for endogenous Nod2 within T cells in protection against uveitis. Nod2-deficient CD4^+^ T cells produce more IL-17 and cause worse uveitis; a mechanism that is independent of Rip2-signaling or microbial stimuli, as MDP or in adjuvant. This T cell-intrinsic function of Nod2 is involved in controlling activation of antigen-experienced CD4^+^ T cells, Th17-associated genes and Ccr7 expression in response to TCR-stimulation, including self-antigen. Moreover, T cells isolated from Blau syndrome patients further underscore how dysregulation of NOD2 results in exacerbated Th17 cells. These data reveal a previously unrecognized role for Nod2 as an inherent T cell-specific modulator of pathogenic Th17 responses that target the eye. Our understanding of how Nod2 contributes to maintenance of healthy T cell responses could yield therapeutic strategies that target NOD2 in T cells to treat Blau syndrome or other NOD2-associated conditions.

## Results

### Nod2 plays an unexpected protective role in uveitis

**Fig. 1:**
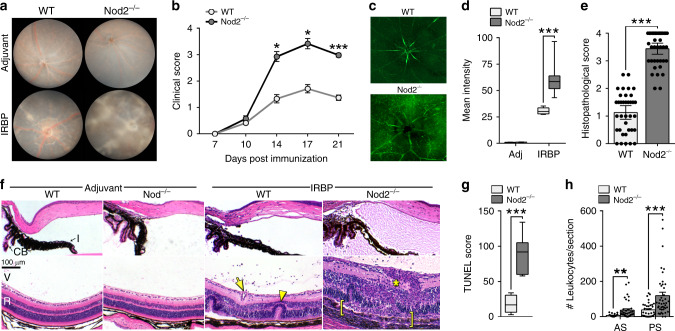
Nod2 plays an unexpected protective role in uveitis. Six to ten week old WT and Nod2^−/−^ mice were immunized and assessed weekly thereafter for disease. **a** Representative fundus images of the posterior pole at day 21 post-immunization, and (**b**) clinical grading of uveitis based on fundoscopy. Data in **b** is combined from 6 independent experiments with an *n* = 12 mice/group for day 7 and day 17, *n* = 24 (WT) and 19 (Nod2^−/−^) mice/group for day 10, *n* = 44 (WT) and 28 (Nod2^−/−^) mice/group for day 14, and *n* = 56 (WT) and 54 (Nod2^−/−^) day 21; *p = 0.0177 (14 day), *p = 0.0093 (day 17), and ***p < 0.0001 (day 21). **c** Representative epifluorescence images and (**d**) quantification of blood-retinal barrier integrity 10 days post-immunization. Data is combined from 2 independent experiments where *n* = 9 (WT adjuvant and Nod2^−/−^ IRBP) or 8 (WT IRBP and Nod2^−/−^ adjuvant) mice; ***p < 0.0001. **e** Uveitis was scored by histopathology 21 days post-immunization. Data was combined from 6 independent experiments where *n* = 38 (WT) and 39 (Nod2^−/−^) mice; ***p < 0.0001. **f** Representative H&E-stained sections of the anterior segment (top) and posterior segment (bottom) of eyes day 21 post immunization. Pathological features indicated: retinal folding (arrowhead), granulomatous formation (asterisk), vasculitis (arrow), and severe photoreceptor damage (bracket). I, iris; CB, ciliary body; V, vitreous; R, retina. **g** Apoptosis within the retina (14 days post immunization) was determined by a TUNEL-based assay. Data was combined from 2 independent experiments where *n* = 8 (WT) and 7 (Nod2^−/−^) mice; ***p = 0.0003. **h** Leukocyte infiltration in the aqueous of the anterior segment (AS) and vitreous of the posterior segment (PS) were enumerated. Data are combined from 6 independent experiments where *n* = 35 (WT AS, WT PS), 39 (Nod2^−/−^AS), 42 (Nod2^−/−^ PS) mice; **p = 0.0011 (AS) and ***p = 0.0005 (PS). Data are mean ± SEM (**b**, **e**, **h**) with dots representing individual values (**e**, **h**) or box-whisker plots with median, 25–75th percentile and min–max range (**d**, **g**). Source data are provided as a Source Data file.

Clinical, histopathological and immunological features of EAU resemble multiple characteristics of human uveitis, such as increased breakdown of the blood-retinal barrier, immune cell infiltration, and the central importance of CD4^+^ effector T cells^17^. WT (C57BL/6J) and Nod2^−/−^ mice were immunized with IRBP and monitored clinically by fundoscopy for development of uveitis (Fig. 1a, b). Based on criteria such as optic nerve head inflammation, retinal and choroidal infiltrates, hemorrhages, and retinal vasculitis (Fig. 1a), Nod2^−/−^ mice developed significantly worse clinical uveitis (Fig. 1b). In addition, more extensive blood–retinal barrier breakdown was detectable 10 days post-immunization, prior to clinical onset of uveitis (Fig. 1c, d). Histopathological evaluation at study termination corroborated the increased severity of uveitis in Nod2^−/−^ mice (Fig. 1e). Pathological features included increased subretinal hemorrhages, perivascular and chorioretinal infiltrates (with granulomatous features akin to uveitis in Blau syndrome), and extensive retinal structural damage (e.g., photoreceptor ablation, diffuse atrophy; Fig. 1f). Apoptotic cell death throughout the retina, indicative of irreversible damage, was also quantified (Fig. 1g). Accumulation of cells and proteinaceous material in the anterior chamber and vitreous cavity (Fig. 1h), which also occurs in patients with uveitis, was greater in Nod2^−/−^ mice. Importantly, Nod2-mediated uveitis was IRBP-dependent, as WT and Nod2^−/−^ mice treated with adjuvant alone showed no detectable pathophysiologic or clinical manifestations of disease (Fig. 1a, d, f). Moreover, Naïve Nod2^−/−^ mice exhibited normal gross anatomic features (Supp. Fig. 1a), inner and outer retinal thickness (Supp. Fig. 1b, c) and visual function (Supp. Fig. 1d, e), thereby ruling out major underlying defects of the eyes or retinae in Nod2^−/−^ mice.

Since host microbiota has been reported to influence T cell development^18^, several Nod2-mediated disease models^9^, as well as pathogenesis of uveitis^19–21^, we sought to determine whether Nod2-mediated uveitis was related to altered microbiome due to different housing conditions. To do this, we compared uveitis development in WT and Nod2^−/−^ mice bred within our facility to newly arrived counterparts shipped directly from The Jackson Laboratory (Supp. Fig. 2a). Mice of a particular genotype were similar to each other despite differences in origin, thereby ruling out differences attributed to colony-specific environments, as well as the possibility of genetic drift in our mouse line. The effect of microbiota in Nod2-mediated uveitis was also evaluated through co-housing studies (Supp. Fig. 2b). Nod2^−/−^ mice that were cohoused 1:1 with WT mice displayed exacerbated uveitis similar to that of non-cohoused Nod2^−/−^ mice, suggesting that the Nod2-mediated phenotype is not influenced by the composition of transmittable microbiota. Further, oral treatment with broad-spectrum antibiotics suppressed induction of EAU in both genotypes (Supp. Fig. 2c), indicating the importance of intestinal microbiota in promotion of EAU as reported^20^. Yet, a significant difference in uveitis severity between antibiotic-treated WT and Nod2^−/−^ mice was still observed.

**Fig. 2:**
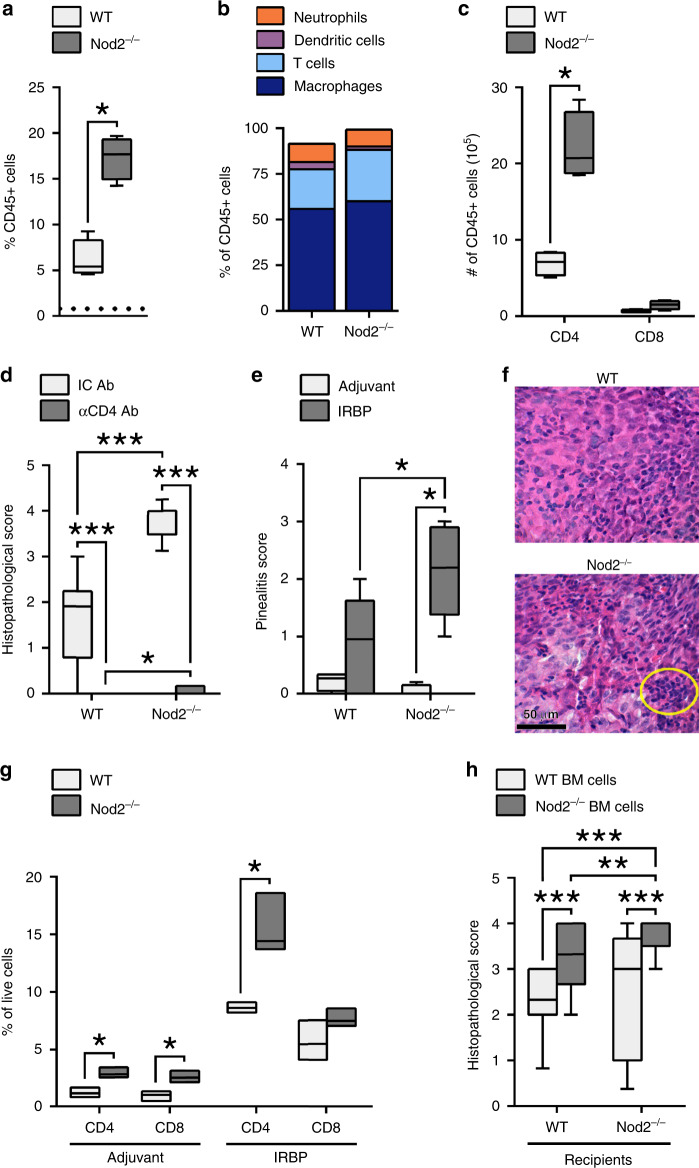
Nod2 influences retinal antigen-specific cellular responses. **a** The proportion of ocular CD45^+^ cells of WT and Nod2^−/−^ mice 20 days post-immunization was determined by flow cytometry. Dashed line denotes baseline level for adjuvant control eyes. **b** Frequency of macrophages (CD45^+^CD11b^+^F4/80^+^), neutrophils (CD45^+^CD11b^+^GR-1^+^), dendritic cells (CD45^+^CD11c^+^), and T cells (CD45^+^CD3^+^CD4^+^) are summarized as stacked bars. **c** Live T cell populations of gated CD45^+^ cells. Data (**a**–**c**) are representative of 4 independent experiments each with *n* = 4 mice/group; *p = 0.0286 (**a**, **c**). **d** Uveitis was evaluated in immunized WT or Nod2^−/−^ mice that were depleted of CD4^+^ T cells with α-CD4 mAb or Isotype control Ab (IC Ab). Data are representative of 2 independent experiments with *n* = 12 mice/group; ***p < 0.0001 (WT IC vs. WT Ab, Nod2^−/−^ IC vs. Nod2^−/−^ Ab, WT IC vs. Nod2^−/−^ IC), and *p = 0.0373 (WT Ab vs. Nod2^−/−^ Ab). **e**–**g** Pinealitis was assessed in WT and Nod2^−/−^ mice 14 days post-immunization. **e** Pinealitis scores from histopathological evaluation, and (**f**) representative H&E-stained sections (circle denotes dense lymphocytic infiltration). Scores in **e** were combined from 2 independent experiments (shown left to right with *n* = 4, 6, 4, 5); *p = 0.0433 (WT IRBP vs. Nod2^−/−^ IRBP), *p = 0.0159 (Nod2^−/−^ adjuvant vs. Nod2^−/−^ IRBP). **g** Frequency of CD4^+^ and CD8^+^ T cells in pineal glands was determined by flow cytometry. Data are combined from 3 independent experiments, each having 5 pooled pineal glands/genotype/treatment. For CD4^+^ cells: *p = 0.0111 (WT IRBP vs. Nod2^−/−^ IRBP) and *p = 0.0081 (WT adjuvant vs. Nod2^−/−^ adjuvant)^.^ For CD8^+^ cells: *p = 0.0122 (WT adjuvant vs. Nod2^−/−^ adjuvant^)^. **h** BM chimeric mice were assessed for EAU by histopathology 20 days post-immunization. Data are combined from 3 independent experiments (shown left to right with *n* = 31,31,30,28); **p = 0.0030 and ***p < 0.0001. Data are box-whisker plots showing median, 25th–75th percentile and min–max range (**a**, **c–e**, **h**), floating bars showing median with min–max range (**g**), or mean (**b**). Data were analyzed with two-tailed Mann–Whitney U test (**a**–**e**, **h**) or unpaired, two-tailed Student’s *t*-test (**g**). Source data are provided as a Source Data file.

### Nod2 influences retinal, antigen-specific cellular responses

Examination of the cellular responses within uveitic eyes of Nod2^−/−^ mice revealed increased infiltration of hematopoietic CD45^+^ cells (Fig. 2a) compared to adjuvant controls (indicated by dotted line; Fig. 2a), which proportionally was comprised primarily of CD11b^+^ macrophages and T cell populations (Fig. 2b). Numerically, eyes from Nod2^−/−^ mice had greater numbers of CD45^+^ cells that were skewed toward the CD4^+^ T population (Fig. 2c), with Nod2^−/−^ mice having a greater CD4:CD8 ratio (15:1) compared to WT (10:1). Importantly, the exacerbated uveitis observed in Nod2^−/−^ mice was CD4^+^ T cell-dependent, as depletion of CD4^+^ T cells ameliorated uveitis in both Nod2^−/−^ mice and WT mice (Fig. 2d).

Ocular immune privilege, which protects the eye from sight-threatening inflammation^22,23^, involves physical sequestration of tissue-specific antigens (TSAs) such as IRBP, in combination with active immunosuppressive mechanisms. Tissue-resident functions of Nod2 within the eye have been described, which could potentiate uveitis^24–26^. Since we have observed Nod2 expression in ocular tissues, most notably within the retina (Supp. Fig. 3), we sought to discern whether Nod2-mediated protection is controlled by antigen-specific versus tissue-specific responses as controlled within the eye. IRBP is not expressed outside of the eye, with the exception being the pineal gland^27^. Thus, we examined the pineal gland, as it is located outside of the blood-ocular barrier and therefore does not benefit from the protective mechanisms of ocular immune privilege. In response to IRBP immunization, but not adjuvant alone, Nod2^−/−^ mice developed a more severe form of pinealitis (Fig. 2e), which was characterized by dense mononuclear cell infiltration and increased areas of cell atrophy (Fig. 2f). As with Nod2-mediated uveitis, the exacerbated pinealitis involved increased CD4^+^ T cells and CD4:CD8 ratio (Fig. 2g); thereby supporting an antigen-specific response by which Nod2 controls uveitis.

**Fig. 3:**
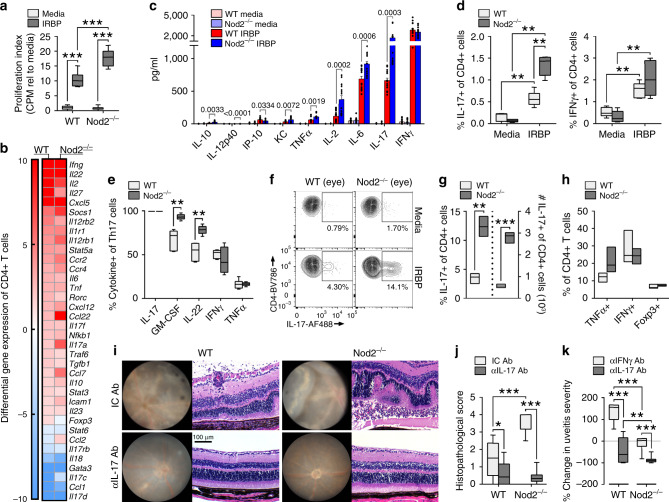
Nod2 negatively regulates hyper-pathogenic Th17 cells. Splenocytes from immunized WT and Nod2^−/−^ mice were stimulated in vitro with IRBP_1–20_ (**a**–**e**). **a** [^3^H] thymidine incorporation after 72 h. Data are combined from 3 independent experiments, *n* = 12 mice/group; ***p < 0.0001. **b** Gene expression of CD4^+^ T cells 18 h was assessed by qPCR. Heatmap indicates expression relative to IRBP_1–20_-stimulated CD4^+^ T cells from adjuvant-control mice. **c** Cytokine production after 48 h was assessed by ELISA. Data are combined from 4 independent experiments, *n* = 16 mice/group. **d** Frequencies of IL-17^+^ or IFNγ^+^ of CD4^+^Thy1.2^+^ T cells was determined by flow cytometry. Data are combined from 2 independent experiments, *n* = 5 (unstimulated) or 6 (IRPB-stimulated) mice/group. IL-17: **p = 0.0043 (WT unstimulated vs. WT peptide, Nod2^−/−^ unstimulated vs. Nod2^−/−^ peptide^),^ **p = 0.0022 (WT peptide vs. Nod2^−/−^ peptide^).^ IFNγ: **p = 0.0043 (WT unstimulated vs. WT peptide), **p = 0.0025 (Nod2^−/−^ unstimulated vs. Nod2^−/−^ peptide^)^. **e** Proportion of Th17 cells (IL-17^+^CD4^+^Thy1.2^+^) co-producing cytokines. Data are combined from 2 independent experiments, *n* = 6 mice/group; **p = 0.0022. **f**–**h** Ocular cells from immunized mice were stimulated in vitro with IRBP_1–20_ for 18 h. Data are combined from 3 independent experiments, *n* = 6 mice pooled/genotype. **f** Representative contour plot showing IL-17^+^CD4^+^ T cells. **g** IL-17^+^ cells from gated live CD4^+^Thy1.2^+^ T cells. ^*^*p = 0.0013 (%), ***p = 0.0001 (#). **h** CD4^+^Thy1.2^+^ T cells co^-^expressing TNFα, IFNγ, or Foxp3. **i**–**k** The effect of cytokine neutralization on uveitis was evaluated clinically and histopathologically 21 days post-immunization. Data are combined from 3 independent experiments, *n* = 18 (WT IC, WT αIL-17), 19 (WT αIFNγ, Nod2^−/−^ IC), 17 (Nod2^−/−^ αIL-1^7^, or 23 (Nod2^−/−^ αIFNγ) mice. **i** Representative images of fundi and H&E-stained retinal sections 21 days post-immunization. **j** Uveitis scores from histopathology. *p = 0.0095 (WT IC vs. WT IL-17 Ab), ***p < 0.0001 (Nod2^−/−^ IC vs. Nod2^−/−^ IL-17 Ab, WT IC vs. Nod2^−/−^ IC). **k** Uveitis scores (from **j**) were normalized to the IC Ab group within each genotype (baseline = 0); **p = 0.0035, ***p < 0.0001. Data are box-whisker plots showing median with 25th–75th percentile and min–max range (**a**, **d**–**e**, **j**, **k**), floating bars showing median with min–max range (**g**, **h**), or mean ± SEM (dots represent individual values) (**c**). Data were analyzed by two-tailed Mann–Whitney U test (**a**, **d**–**e**, **h**, **j**) or unpaired, two-tailed Student’s *t*-test (**c**, **g**, **k**). Source data are provided as a Source Data file.

To further assess Nod2 control of any tissue-resident functions, chimeric mice were generated by transplantation of bone marrow (BM)-cells from either WT or Nod2^−/−^ donors (CD45.2) into irradiated congenic WT or Nod2^−/−^ recipients (CD45.1; Fig. 2h). WT recipients transplanted with Nod2^−/−^ BM-cells showed increased susceptibility to EAU (Fig. 2h), comparable to Nod2^−/−^ controls. Conversely, Nod2^−/−^ recipients transplanted with WT BM cells developed less severe uveitis, similar to WT controls. Collectively, these data (Fig. 2) demonstrate that the protective mechanism conferred by Nod2 is mediated by an immune/hematopoietic cellular mechanism involving IRBP-reactive CD4^+^ T cells, and is not likely controlled by a local immunosuppressive function of Nod2 within the eye.

### Nod2 negatively regulates hyper-pathogenic Th17 cells

Based on the findings above, we sought to further evaluate how Nod2 controls antigen-specific T cell responses. IRBP-stimulated Nod2^−/−^ splenocytes were hyper-proliferative (Fig. 3a), indicative of expansion of IRBP-specific T cells. Since both Th1 and Th17 subsets can contribute to EAU^28^ we considered whether Nod2-mediated protection against uveitis involved regulation of a specific Th cell subset. Examination of the Th-associated profile of CD4^+^ T cells purified from immunized WT or Nod2^−/−^ mice revealed increased expression of *Il17a*, as well as other Th17-associated genes (Fig. 3b); whereas other Th1-associated genes such as *Ifnγ* were unchanged. Accordingly, IRBP-stimulated Nod2^−/−^ T cells secreted greater levels of IL-17 (Fig. 3c), but similar levels of the Th1-associated cytokine IFNγ, as WT T cells as measured by ELISA.

When IRBP-specific Th17 responses were evaluated by intracellular cytokine staining (Fig. 3d), peripheral expansion of the Th17 population was observed in Nod2^−/−^ mice. Proportionally, a vast majority of the gated-Th17 population of Nod2^−/−^ Th17 cells co-expressed cytokines GM-CSF and IL-22 (Fig. 3e), which have also been reported to have pathogenic roles in uveitis^29,30^. Whereas gated-Th17 cells of WT and Nod2^−/−^ mice showed comparable frequencies of IFNγ or TNFα co-producers. These data support Nod2 control in expansion of a pathogenic Th17 population. In uveitic eyes of Nod2^−/−^ mice, an enhanced Th17 response was also observed (Fig. 3f, g), which was not associated with altered Th1 or Foxp3^+^ Treg populations (Fig. 3h or transcriptionally as in Fig. 3b). The in vivo contribution of Th17-immunity (over Th1-immunity) in Nod2-mediated uveitis was confirmed by neutralization studies (Fig. 3i–k). Neutralization of IL-17 reduced uveitis severity in both WT and Nod2^−/−^ mice (Fig. 3i, j), albeit to a greater extent proportionally in Nod2^−/−^ mice (Fig. 3k). Whereas neutralization of IFNγ had minimal effect on uveitis severity in Nod2^−/−^ mice, it exacerbated uveitis in WT mice (Fig. 3k; Supp. Fig. 4). Collectively, these observations reveal a causal role for expansion of a pathogenic Th17-response in uveitis of Nod2^−/−^ mice.

**Fig. 4:**
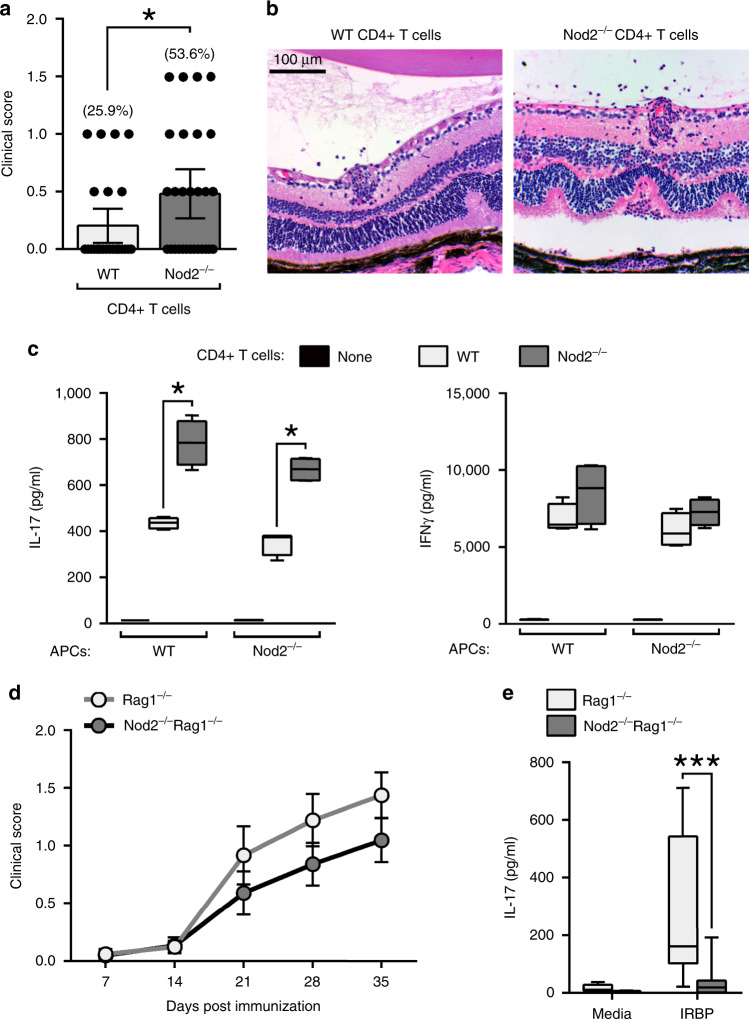
Nod2 suppresses uveitis through a non-conventional cellular mechanism. **a**–**b** CD4^+^ T cells from IRBP-immunized donor WT or Nod2^−/−^ mice were adoptively transferred into naïve WT recipients. **a** Clinical uveitis scores and incidence 20 days post-transfer. Data are from 6 independent experiments with *n* = 27 (WT) and 28 (Nod2^−/−^) mice; *p = 0.0336. **b** Representative photos of WT recipients of WT or Nod2^−/−^ CD4^+^ T cells 20 days post-transfer. **c** IL-17 and IFN-γ were measured by ELISA following 18 h stimulation with IRBP_1–20_ (20 µg/ml) of co-cultured APCs (isolated from naïve WT or Nod2^−/−^ mice) and CD4^+^ T cells (isolated from WT or Nod2^−/−^ immunized mice). Control APCs without the addition of T cells (“none”) were stimulated with IRBP. Data are combined from 4 independent experiments. For each experiment, APCs or T cell populations were isolated from *n* = 3 pooled mice/genotype; *p = 0.0286 for comparisons with both APC conditions. **d**–**e** Rag1^−/−^ or Nod2^−/−^Rag1^−/−^mice were reconstituted with WT CD4^+^ T cells and immunized with IRBP 24 h later. **d** Clinical uveitis was evaluated weekly. Data is combined from 3 independent experiments. For Rag1^−/−^ recipients *n* = 16 (7 days), 20 (14 days), 18 (21 and 28 days), 16 (35 days). For Nod2^−/−^Rag1^−/−^ recipients *n* = 20 (7 days), 22 (14, 21, and 28 days), 20 (35 days). **e** Splenocytes were stimulated 18 h in vitro with IRBP_1–20_ and IL-17 in culture supernatants was measured by ELISA. Data are combined from 3 independent experiments with *n* = 12 (Rag1^−/−^ media), 18 (Nod2^−/−^Rag1^−/−^ media), 13 (Rag1^−/−^ IRBP), 15 (Nod2^−/−^Rag1^−/−^ IRBP) mice; ***p = 0.0001. Data are mean ± SEM (**a**, **d**) with dots representing individual values (**a**) or box-whisker plots with median, 25–75th percentile and min–max range (**c**, **e**). Statistical differences between groups were calculated using unpaired, two tailed Student’s *t*-test (**a**), two-tailed Mann–Whitney U test (**c**, **d**, **e**). Source data are provided as a Source Data file.

### Nod2 inhibition of Th17-mediated uveitis is T cell-intrinsic

Our data thus far support a hematopoietic origin for the cellular mechanism by which Nod2 suppresses IRBP-induction of uveitis, which involves control over CD4^+^ T cells that cause organ-specific autoimmunity. Thus, we next aimed to discern indirect effects of Nod2 within APCs from direct T cellular functions of Nod2 within T cells. Adoptive transfer studies showed that IRBP-reactive Nod2^−/−^ T cells were more pathogenic as they had enhanced ability to trigger uveitis in naïve WT hosts (Fig. 4a, b). Autologous co-culture experiments were performed wherein CD4^+^ T cells purified from IRBP-immunized mice were stimulated in vitro with IRBP in the presence of APCs derived from naive WT or Nod2^−/−^ mice (Fig. 4c). Regardless of APC-genotype, IRBP-specific IL-17 production in Nod2^−/−^ CD4^+^ T cells was augmented. IRBP stimulation of APCs of either genotype (in the absence of co-cultured T cells) did not result in cytokine production; thereby verifying the antigen-T cell specificity of the response measured in this assay. As observed in Fig. 3, IRBP-induced production of IFNγ by CD4^+^ T cells of both genotypes was similar. These data support a direct role of Nod2 within CD4^+^ T cells in suppression of a hyper-pathogenic IRBP-reactive Th17 population during uveitis.

Since a potential caveat for the two studies above (Fig. 4a–c) relates to how T cells may have been polarized during the initial immunization within Nod2^−/−^ mice, we proceeded to evaluate the in vivo contribution of host-derived Nod2 on T cell function. Lymphopenic Rag1^−/−^ mice were crossed with Nod2^−/−^ mice to generate double-knockout mice (Nod2^−/−^Rag1^−/−^ mice). CD4^+^ T cells derived from naïve WT mice were transferred into Rag1^−/−^ or Nod2^−/−^Rag1^−/−^ recipients and T cell reconstitution was verified. After immunization, Rag1^−/−^ and Nod2^−/−^Rag1^−/−^ recipients developed similar onset and severity of clinical uveitis (Fig. 4d), though a trend of reduced severity of uveitis was noted in Nod2^−/−^Rag1^−/−^ recipients. In addition, T cells derived from immunized Nod2^−/−^Rag1^−/−^ hosts had significant reduction in IL-17 production to IRBP stimulation (Fig. 4e), supporting the conventional role ascribed to Nod2 within APCs^3,10^ in promotion of Th17 differentiation. These observations highlight how Nod2 serves multiple, mutually exclusive functions that may have opposing effects on disease. Together, these data reveal a role for Nod2 in control over hyper-inflammatory Th17 cells and suppression of uveitis regardless of host-generated Nod2 or its cellular function in APCs.

**Fig. 5:**
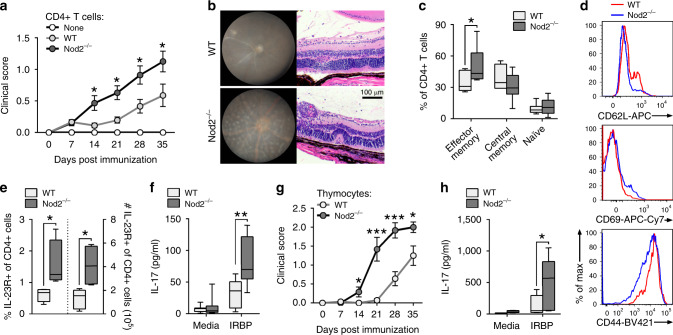
Nod2 inhibition of Th17-mediated uveitis is T cell-intrinsic. Rag1^−/−^ mice were reconstituted with CD4^+^ T cells from naïve WT or Nod2^−/−^ mice and immunized 1 day later (**a**–**f**). **a** Clinical uveitis was evaluated weekly by fundoscopy after immunization. Data is combined from 6 independent experiments and (from left to right), *n* = 43, 40, 38, 24, 17 (WT) and *n* = 30, 46, 46, 28, 12 (Nod2^−/−^) mice; *p = 0.0275 (14 day), *p = 0.0160 (21 day), *p = 0.0216 (28 day), *p = 0.0232 (35 day). **b** Images of fundus and H&E-stained retinal sections 35 days post-immunization. Splenocytes harvested 35 days post-immunization were analyzed by flow cytometry (**c**–**e**) or ELISA (**f**). **c** Frequencies of effector memory (CD62L^lo^CD44^mid^), central memory (CD62L^lo^CD44^hi^), and naïve CD4^+^ T cells (CD62L^hi^CD44^lo^). Data are combined from 2 independent experiments for n = 7 (WT) and 9 (Nod2^−/−^); *p = 0.0418. **d** Cell surface expression of CD62L, CD69, and CD44 on CD4^+^ T cells, and (**e**) frequency and total number of CD4^+^ T cells expressing IL-23R recovered from spleens of IRBP-immunized Rag1^−/−^ recipients. Data are combined from 2 independently performed experiments with *n* = 5 (WT) and n = 4 (Nod2^-/-^) mice; *p = 0.0159 (% and #). **f** IL-17 production was measured by ELISA following 18 h in vitro stimulation with IRBP_1–20_. Data are combined from 2 independent experiments. For WT, *n* = 10 (media) and 9 (peptide) and for Nod2^−/−^, *n* = 11(media) and 10 (peptide) mice; **p = 0.0030. (**g**-**h**) Rag1^−/−^ mice were reconstituted with thymocytes derived from naïve WT or Nod2^−/−^ mice and immunized with IRBP 8 days later. **g** Clinical uveitis was evaluated weekly by fundoscopy. Data is combined from 2 independent experiments. For WT, *n* = 14 (7, 14, 21, 35 day), 13 (28 day) and for Nod2^−/−^, *n* = 12 mice; *p = 0.0331 (14 day), ***p < 0.0001 (21 day), ***p = 0.0002 (28 day), *p = 0.0347 (35 day). **h** IL-17 production was measured with ELISA following 18 h in vitro stimulation of splenocytes with IRBP_1–20_. Data are combined from 2 independent experiments with *n* = 9 (WT media) and 8 (all other conditions) mice; *p = 0.0281. Data are mean ± SEM (**a**, **g**), box-whisker plots showing median, 25–75th percentile and min-max range (**c**, **f**), or box-whisker plots showing median, 10–90th percentile and min-max (**e**, **h**). Statistical differences between groups were calculated using two-tailed Mann–Whitney U test. Source data are provided as a Source Data file.

The studies above excluded any host cellular function for Nod2 in suppression of uveitis. We next directly evaluated the T cell-intrinsic function for Nod2. CD4^+^ T cells derived from naïve WT or Nod2^−/−^ mice were transferred into Rag1^−/−^ recipients (Fig. 5a–d). In response to immunization, Rag1^−/−^ recipients of Nod2^−/−^ CD4^+^ T cells showed significant expansion of CD4^+^ T cells (Supp. Fig. 5), which correlated with earlier onset and more severe uveitis compared to recipients of WT CD4^+^ T cells (Fig. 5a, b). In subtle contrast to mice immunized directly for EAU, immunized reconstituted Rag1^−/−^ recipients often developed multiple discrete retinal inflammatory lesions with little prior involvement of the optic nerve and retinal vasculature (Fig. 5b). Interestingly, uveitis in Blau syndrome has been described as having multiple, non-coalescing and nodular chorioretinal foci surrounding the optic disc^5^. Once established, the exacerbated uveitis mediated by Nod2^−/−^ CD4^+^ T cells was characterized histopathologically by focal granulomatous inflammation, retinal folding, and vasculitis (Fig. 5b). Control Rag1^−/−^ mice lacking CD4^+^ T cells did not develop uveitis after immunization (Fig. 5a), confirming the requirement for CD4^+^ T cells in this model. We also observed a T cell-intrinsic function for Nod2 in suppression of the IRBP-induction of effector memory (CD62L^lo^CD44^mid^) CD4^+^ T cells (Fig. 5c) and T cell activation, as Nod2^−/−^ CD4^+^ T cells expressed relatively lower CD62L and CD44 with higher CD69 (Fig. 5d).

We next examined the T cell-intrinsic role for Nod2 in control of Th17-immunity (Fig. 5e, f). The proportion and number of Nod2^−/−^ CD4^+^ T cells expressing IL-23R, a critical upstream inducer of IL-17 within T cells, were increased compared to WT CD4^+^ T cells in reconstituted Rag1^−/−^ mice (Fig. 5e). Nod2^−/−^ CD4^+^ T cells primed in Rag1^−/−^ hosts also produced greater amounts of IL-17 in response to IRBP-stimulation in vitro (Fig. 5f). Lastly, an independent approach to test the T cell-autonomous function of Nod2 in uveitis was undertaken using naïve thymocytes in adoptive transfer studies with Rag1^−/−^ hosts. Thymocytes from WT and Nod2^−/−^ mice, which are known to have similar cellular composition^31^, were adoptively transferred into Rag1^−/−^ recipients (Fig. 5g, h) and similar reconstitution levels were verified prior to immunization. Similar to peripherally-derived CD4^+^ T cells (Fig. 5a, b), Nod2^−/−^ thymocytes elicited a more severe uveitis compared to WT thymocytes (Fig. 5g), and recapitulated the T cell-intrinsic signature of augmented IL-17 production to IRBP-stimulation in vitro (Fig. 5h). Collectively, these data demonstrate an essential T cell-autonomous role for Nod2 in suppression of antigen-specific uveitogenic Th17 cells, which is remarkably inherent to CD4^+^ T cells rather than related to peripheral regulatory mechanisms.

### Nod2 functions independent of Rip2-signaling in T cells

**Fig. 6:**
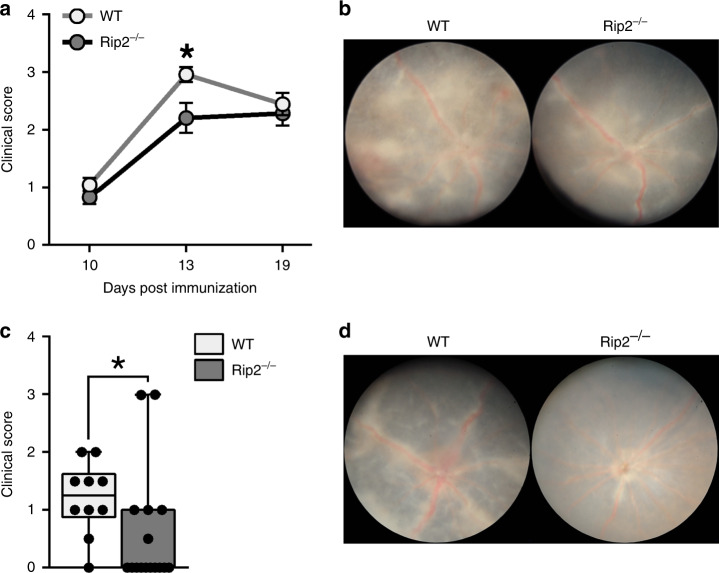
Nod2 functions independent of Rip2-signaling in T cells. **a**–**b** EAU was induced in WT and Rip2^−/−^ mice. Data are combined from 3 independent experiments. For WT, *n* = 10 (10 and 19 day) and 24 (13 day). For Rip2^−/−^, *n* = 9 (10 day), 21 (13 day), 12 (19 day). **a** Clinical uveitis was evaluated weekly by fundoscopy; *p = 0.0320. **b** Representative fundus photographs 13 days post-immunization. **c**–**d** Rag1^−/−^ mice were reconstituted with thymocytes from naïve WT or Rip2^−/−^ mice and immunized with IRBP 8 days later. Data are combined from 2 independent experiments with *n* = 10 (WT recipients) and *n* = 18 (Rip2^−/−^ recipients). **c** Clinical uveitis was evaluated by fundoscopy 28 days post-immunization; *p = 0.0074. **d** Representative fundus photographs are shown. Data are mean ± SEM (**a**) or box-whisker plots showing median, 25–75th percentile, and min–max range (with dots representing individual values) (**c**). Statistical differences between groups were calculated using two-tailed Mann–Whitney U test (**a**, **c**). Source data are provided as a Source Data file.

As an innate immune receptor that senses MDP, most cellular functions of Nod2 are attributed to Rip2-signaling. Thus, we evaluated whether the T cell-intrinsic mechanism by which Nod2 suppresses uveitis involved the canonical Rip2-signaling axis. Uveitis in global Rip2^−/−^ mice was attenuated at the peak of disease (d 13) compared to WT mice (Fig. 6a, b), but reached similar uveitis severity by d 19 (Fig. 6a). This supported a role for Nod2 that was not analogous to that of Rip2 in regulation of uveitis. The Rip2-signaling axis was then directly evaluated within T cells during uveitis. To focus on a T cell-inherent function of Rip2 without effects of Rip2-deficiency on peripheral tolerance mechanisms, we conducted thymocyte transfer studies as in Fig. 5. After immunization of Rag1^−/−^ recipients, the transferred Rip2^−/−^ thymocytes (which have similar composition to WT mice^32^) elicited significantly less severe uveitis compared to WT thymocytes (Fig. 6c, d), indicating an inherent and opposing T cellular function for Rip2 in pathogenesis of uveitis. Collectively these data reveal a unique “uncoupling” of the classically described Nod2/Rip2-signaling axis in T cells.

### Nod2 controls Th17 immunity in naïve mice and Blau syndrome

**Fig. 7:**
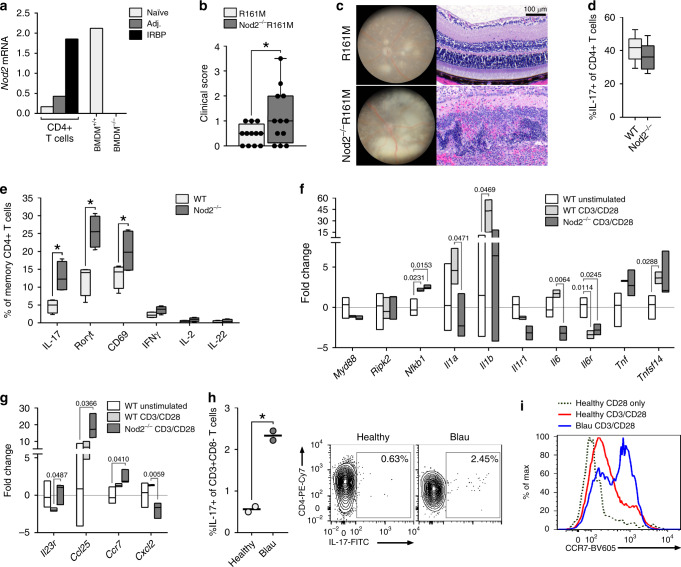
Nod2 controls Th17 immunity in naïve mice and Blau syndrome. **a** *Nod2* expression in IRBP_1–20_–stimulated CD4^+^ T cells of naïve, adjuvant-only, or immunized WT mice relative to unstimulated CD4^+^ T cells from naïve WT mice was measured by qPCR. Controls include IFNγ-stimulated WT BMDM and Nod2^−/−^ BMDM cells. Data are representative of 2 independent experiments, each with *n* = 5 spleens pooled/genotype. **b**–**c** Rag2^−/−^ hosts reconstituted with CD4^+^ T cells from naïve R161M or Nod2^−/−^R161M mice were assessed for uveitis 7 days post-transfer. Data are combined from 3 independent experiments with *n* = 12 mice/group. **b** Clinical uveitis was scored, *p = 0.0500. **c** Representative fundus and histopathology photographs. **d**–**g** T cell responses in naïve WT and Nod2^−/−^ (C57BL/6 J) mice. **d** Naïve CD4^+^ T cells (CD62L^hi^CD44^-^) from WT and Nod2^−/−^ mice were differentiated under Th17-polarizing conditions and frequency of IL-17-producing CD4^+^ T cells (CD4^+^CD8^-^Thy1.2^+^IL-17^+^) was determined by flow cytometry. Data are combined from 2 independent experiments for *n* = 8 mice/genotype. **e** Frequency of memory (CD62L^-^CD44^+^) CD4^+^ T cells from naïve WT or Nod2^−/−^ mice expressing indicated factors was determined by flow cytometry 5 days post-stimulation with anti-CD3/anti-CD28 mAb. Data is representative of 3 independently performed experiments, each with *n* = 4 mice/genotype; *p = 0.0286 for all indicated comparisons. **f**–**g** Memory CD4^+^ T cells from naive WT or Nod2^−/−^ mice stimulated 16 h with anti-CD3/anti-CD28 mAb were analyzed by multiplex-qPCR. Transcripts are relative to unstimulated control memory CD4^+^ T cells of WT mice (horizontal line), with *n* = 3 mice/condition. **h**–**i** PBMCs of two healthy controls or two Blau syndrome patients were stimulated with anti-CD3/anti-CD28 mAb for 5 days then with PMA/ionomycin for 4 h. Experiment was repeated twice. **h** Frequency of IL-17-producing CD4^+^ T cells (CD3^+^CD4^+^CD19^-^IL-17^+^)(*p = 0.0055) and representative contour plot. **i** Representative histogram showing MFI of CCR7 by CD4^+^ T cells (CCR7^+^CD3^+^CD4^+^CD19^-^). Data are box-whisker plots showing median with 25–75th percentile and min–max range (**b**, **d**), box-whisker plots showing median with 10–90th percentile (**e**), or floating bars showing median and min–max range (**f**, **g**). Dots represent individual values (**b**, **h**). Statistical differences were calculated using two-tailed Mann–Whitney U test (**d**, **e**) or unpaired, two-tailed Student’s *t*-test (**b**, **f**–**h**). Source data are provided as a Source Data file.^+^.

Given that the T cell-intrinsic function of Nod2 appeared to occur in a Rip2-independent manner, we turned to investigate how Nod2 controls antigen-triggered T cell responses downstream of the TCR. Indeed, *Nod2* transcription was markedly upregulated in WT CD4^+^ T cells stimulated with IRBP, to a level comparable to that of activated BM-derived macrophages that constitutively expresses *Nod2*; thereby indicating a direct response between antigen recognition and *Nod2* expression (Fig. 7a). To examine how Nod2 expression alters antigen-specific TCR function in vivo, we crossed IRBP TCR-Tg mice (i.e., R161M mice on the B10.RIII background^33^) with Nod2^−/−^ mice (on B10.RIII background) to generate Nod2^−/−^R161M mice. In R161M mice, the CD4^+^ T cells recognize IRBP via a transgenic αβ TCR and develop spontaneous uveitis without immunization. Importantly, their T cells are capable of inducing uveitis after adoptive transfer into lymphopenic recipients (i.e., without prior in vitro activation)^33^. CD4^+^ T cells purified from naïve R161M mice or Nod2^−/−^R161M mice were transferred into naïve syngeneic B10.RIII Rag2^−/−^ mice that were monitored for development of uveitis (Fig. 7b, c). Recipients of Nod2^−/−^R161M CD4^+^ T cells developed more severe uveitis than those of R161M CD4^+^ T cells (Fig. 7b, c). These data underscore the importance a T cell-autonomous function of Nod2 in control over the pathogenic potential of autoreactive and/or antigen-specific T cell responses. These data also provide evidence that the T cell-intrinsic mechanism of Nod2 does not require IRBP-immunization in the context of CFA. In support of the latter, we have also found that Nod2^−/−^ mice immunized with non-CFA or MDP-free adjuvants (e.g., heat-killed *S. cerevisiae* or *C. albicans*) develop exacerbated uveitis compared to WT mice (Supp. Fig. 6a). Moreover, IRBP TCR Tg T cells, which produce IL-17, also failed to produce heightened IL-17 in response to MDP exposure in vitro (Supp. Fig. 6b). Collectively, these data support a unique T cell-autonomous function for Nod2 in TCR signaling induced by peptide recognition that is independent of established tenets of Nod2 biology pertaining to MDP sensing or Rip2-signaling.

Since Nod2 acts as a molecular modulator of deleterious IRBP-autoreactive T cell responses, we next considered whether it might also influence general TCR functions under homeostatic conditions. All in vitro studies were carried out in serum-free conditions, given that serum can be contaminated with trace amounts of PGN/MDP^34^. To do this, we examined the function of Nod2 downstream of TCR-ligation in naïve mice (Fig. 7d–g). To determine whether Nod2 expression controlled conventional differentiation of naïve CD4^+^ T cells into Th17 cells, naïve (CD62L^hi^CD44^−^) CD4^+^ T cells isolated from unimmunized WT or Nod2^−/−^ mice were stimulated in vitro with CD3/CD28 under Th17-polarizing conditions (Fig. 7d). WT and Nod2^−/−^ T cells had equal capacity to differentiate into Th17 cells, indicating that Nod2 is dispensable for in vitro Th17 differentiation of naïve CD4^+^ T cells.

A critical aspect of maintaining homeostasis of T cells is constant low-affinity interactions between TCR and cognate self-antigen/MHC ligands^35^. During homeostatic expansion, naïve T cells can acquire phenotypic and functional properties of antigen-experienced (or memory) T cells, a critical process that can also enhance autoimmune T cell-responses^36^. Given the connection between induction of Nod2 with cognate-TCR ligation (Fig. 7a) and its function in suppression of autoreactive T cells (Fig. 7b–c), coupled with a prior report showing that Nod2 is preferentially expressed in antigen-experienced (memory) T cells^12^, we considered a Nod2-specific function in the memory (CD62L^−^CD44^+^) CD4^+^ T cell population. Indeed, when memory CD4^+^ T cells isolated from naive WT vs. Nod2^−/−^ mice were activated with CD3/CD28, a significantly greater proportion of Nod2^−/−^ T cells produced IL-17 and the IL-17-associated transcription factor RORγt (Fig. 7e). CD69 was also increased in Nod2^−/−^ memory T cells, indicative of a role for Nod2 in control of activation state (Fig. 7e). A more detailed analysis of earlier TCR signaling events controlled by Nod2 specifically within memory T cells of unimmunized mice revealed a transcriptionally-distinct population to WT memory T cells (Fig. 7f, g). In response to CD3/CD28 stimulation, expression of signaling mediators and/or cytokines typically induced by the canonical Rip2/NF-kB pathway remained unchanged (e.g., *Myd88*, *Ripk2*, *Tnf*) or even reduced (e.g., *Il1a*, *Il6*) by Nod2-deficiency (Fig. 7f), further supporting a non-canonical signaling-function of Nod2 in T cells. However, four T cell factors not typically associated with Rip2-signaling were found to be uniquely and significantly regulated by Nod2^−/−^ memory T cells; *Il23r*, *Ccl25*, *Ccr7*, and *Cxcl2*, all of which participate in controlling key T cell processes such as Th17 differentiation, T cell development, and co-stimulation (Fig. 7g). Collectively, these data identify Nod2 as a homeostatic factor in the integration of signals that control generation of antigen-experienced uveitogenic Th17 cells.

Our findings demonstrated that uveitis arises from inherent T cell dysfunction as a consequence of loss-of-function of Nod2. This is contrary to the long-standing hypotheses pertaining to the pathogenesis of Blau syndrome that propose that gain-of function mutations result in overactive Nod2 and promotion of the canonical Rip2-pathway in innate myeloid cells. Thus, we directly tested for defects in T cellular production of IL-17 in two related patients with Blau syndrome (heterozygous for CARD15 c.1147G->A mutation, leading to pGlu383Lys) (Fig. 7h, i). Despite differences in age, gender, and treatments these patients possessed similar and significantly greater proportions of Th17 cells compared to healthy controls (Fig. 7h). As with Nod2^−/−^ memory T cells in mice, which showed increased expression of Ccr7 in response to TCR-ligation (Fig. 7g), CD3/CD28 stimulation also increased expression of CCR7 on CD4^+^ T cells similarly in both the patients with Blau syndrome compared to healthy controls (Fig. 7i). CCR7 is an integral molecule in co-stimulation and the establishment of T cell tolerance, as it has been recently described as a marker for autoreactive T cells^37^. Thus, our data suggest a previously unappreciated T cell defect in Blau syndrome as a consequence of NOD2-dysfunction.

## Discussion

A prevailing theory proposes that uveitis involves innate signals arising from early microbial-genetic interactions that engender a break in T cell tolerance. We stand to gain important insight from the study of the innate immune receptor Nod2, as it exists at the juxtaposition of microbial-host responses and uveitis, wherein mutation of NOD2 causes uveitis and arthritis (i.e., Blau syndrome). While the role for Nod2 in promotion of Th-immunity is well-known^8^, our work extends the previously ascribed antimicrobial, MDP-sensing function of Nod2 in APCs and Th-priming, to also include one as a T cell-intrinsic modulator of the TCR-signaling axis in autoreactive Th17 cells that cause uveitis. Prior evaluations of direct functions for Nod2 in T cells have yielded confounding results^12,13,16^. Initially, Nod2-deficiency was reported to increase susceptibility to *Toxoplasma gondii* infection, owing to a T cell-intrinsic defect in Th1 immunity and IL-2 production^16^. These results were contradicted by a subsequent report showing that Nod2-deficiency was dispensable in *T. gondii* infection, Th1 differentiation and IL-2 production^13^; thereby placing a T cell-intrinsic role for Nod2 into question. Here, our work provides evidence for an integral regulatory role for Nod2 in suppression of the TCR-signaling axis in uveitis as might relate to Blau syndrome.

We would posit that the pleiotropic functions of Nod2 may be explained based on T cell-specific roles in antigen recognition vs. microbial sensing functions that help maintain balance between self-tolerance and host-defense mechanisms against infection, respectively. Our results are further supported by recent observations of similar discordant Nod2-Rip2 signaling functions in T cell responses; specifically, induction of c-Rel activity and induction of IL-2 to promote Th1 immunity^16^, CD8^+^ T cell development^32^, and CD69 expression and activation-induced cell death in response to alloantigen as is relevant to graft-versus-host-disease^14^. Thus, our work complements other studies in which Rip2 also was found to be dispensable for T cell function^14,32,38,39^. In this regard, one appreciates that due to dueling or compensatory cell-specific functions of Nod2, a T cell-specific role may be masked in global Nod2^−/−^ mice (where all cells lack Nod2); a confound that may have hindered full understanding of the involvement of Nod2 in infection and inflammatory diseases.

Our data reveal a role for Nod2 downstream of the TCR complex and/or co-stimulatory molecules (i.e., CD28 and B7), supporting a role for Nod2 in response to antigen stimulation. This would be supported by the prior report that Nod2 controlled OT-II Tg T cell responses to OVA^16^. In WT CD4^+^ T cells, Nod2 has also been shown to be induced by TCR-ligation using 10 μg/ml anti-CD3 and 2 μg/ml anti-CD28^12^, but not when T cells are stimulated with lesser amounts of anti-CD3 (e.g., 2 or 5 μg/ml) with 2 μg/ml anti-CD28^12,14^. Intriguingly, Nod2-deficient T cells stimulated with the lower doses of CD3/CD28 (2 μg/ml) also exhibit normal responses such as T cell activation, proliferation or cytokine production^13^, suggesting that a role for Nod2 in response to antigen occurs in a dose-dependent manner. Likewise, in an in vivo model of T cell-triggered enteropathy, injection of 50 μg anti-CD3 in Nod2^−/−^ mice elicited an exacerbated Th17-response and intestinal disease^40^, whereas lower doses of anti-CD3 (15 μg) did not^41^. Cumulatively, these data suggest that Nod2 modulates antigen-induced T cell responses, which may be influenced by TCR-signal strength and/or TCR-affinity.

Our data further reveal a homeostatic function of Nod2 specific to memory CD4^+^ T cells. Constitutive expression of Nod2 in antigen-experienced (i.e., memory CD44^+^) CD4^+^ T cells has been reported^12^, but its functional role in this T cell subset has not been described. We show a proximal role for Nod2 in shaping TCR-signaling responses towards Th17-immunity. For example, Ccr7 is known to participate in T cell-homeostasis and trafficking^42^ and Ccr7 activation by its agonist Ccl21 directly amplifies antigen-specific T cell responses and T cell homeostatic proliferation^43^. Such functions would be relevant to autoimmunity, as Ccr7 promotes Th17-mediated autoimmune disease in experimental dry eye disease^44^, multiple sclerosis (EAE)^45^, and arthritis^46^. Thus, Nod2 may function similar to a co-stimulatory factor to temper the TCR-signaling threshold in order to control antigen-experienced T cells that are self-reactive pathogenic Th17 cells. Indeed, memory T cells have less stringent requirements for activation (i.e., cytokines only) compared to naïve T cells that require continuous low-affinity TCR interactions with self-antigen for survival under homeostatic conditions^35^, further supporting Nod2 as a check-point inhibitor of downstream antigen-specific T cell activation. This is in line with a prior report showing that Nod2 acts downstream of CD28^16^, which is of interest since B7 is known to fine-tune antigen-specific CD4^+^ T cell responses^47^, including suppression of Th17 differentiation^48^.

Finally, our work suggests that pathology of Blau syndrome may not be driven solely by inflammatory microbial signals as controlled by myeloid cells, but could also include dysregulated T cell responses. Since first being described phenotypically^6,7^ and genetically^4^, Blau syndrome has been classified as the “prototypic autoinflammatory” disease^49^. This is based on the apparent lack of high titer auto-antibodies (e.g., RF, ANA, ANCA) and phenotypic similarities with other NLR-mediated autoinflammatory diseases, which are presumed to be due to hyper-inflammatory responses such as excessive IL-1β production by innate myeloid cells. Indeed, early studies in HEK cell lines transfected with NOD2 or Blau-variant NOD2 showed excessive NF-κB activity^50,51^ at baseline levels and in response to MDP; thereby implicating over-activation of the canonical Nod2-signaling axis in Blau syndrome. However, more recent in vitro studies using PBMCs or monocytes^52–55^ derived from patients with Blau syndrome showed reduced NF-κB activation and cytokines such as IL-1β in response to MDP, suggestive of a loss-of-function or “exhausted phenotype” in myeloid cells. We have also observed impaired MDP-signaling and NF-κB activation in macrophages derived from Blau patients, a cellular mechanism which was further dissected in Blau-mutant mice^56^. Thus, the idea of how gain-of-function of NOD2 causes Blau syndrome has been challenged. Our data suggest that hyper-pathogenic Th17 cells arising from dysfunctional Nod2 could relate to Blau pathology. We showed that T cells from patients with Blau syndrome produce excessive IL-17 and over express CCR7 upon TCR-ligation, thereby paralleling the hyper-pathogenic T cell responses found in Nod2^−/−^ mice. While the patient sample number was small for this especially rare disease, these findings are supported by reports of Blau syndrome patients who had increased effector/memory CD4^+^ T cells within eyes^57^ and in skin granulomas, which also coincided with increased IL-17 and IL-23R immunoreactivity^58^. Taken together, these data present an alternative to the current perspective that Blau syndrome represents an autoinflammatory disease caused by a gain-of-function of Nod2. In conclusion, that Nod2 functions as an endogenous protectant against uveitis has clear implications for development of therapeutic strategies to correct underlying T cell defects in uveitis and other forms of autoimmunity.

## Methods

### Mice

C57BL/6J (Stock # 000664), Nod2^−/−^ (Stock # 005763), Rip2^−/−^ (Stock # 007017) and Rag1^−/−^ (Stock #002216) mice were purchased from The Jackson Laboratory (Bar Harbor, ME, USA) and maintained as colonies in-house. Rag1^−/−^ mice (on C57Bl/J strain) were crossed with Nod2^−/−^ mice to generate double Nod2^−/−^Rag1^−/−^ mice. Since genetically-modified mice derived from 129ES cells (i.e., Nod2^−/−^ and Rip2^−/−^ mice) may contain an inactivating Caspase-11 mutation^59^, we confirmed by genotyping that all of these lines: WT, Nod2^−/−^ and Rip2^−/−^ mice possessed the WT form of Caspase-11 (CTCTCTTCACAGATCACTTGTCCTA). R161M mice that express a transgenic T cell receptor specific for IRBP_161–180_ and Rag2^−/−^ mice, both on B10.RIII background, have been described^33^. To generate Nod2^−/−^R161M mice, Nod2^−/−^mice were backcrossed in-house at least 10 generations onto B10.RIII (Jackson Stock # 000457), after which they were crossed with R161M mice. Experimental and control mice were bred separately in specific pathogen-free (SPF) conditions at the VA Portland Health Care System, where they were maintained at 21 °C on a 12 h light-dark cycle (6 a.m. to 6 p.m.) and given free access to food and water. As we do not observe any sex-bias for males vs. females for the Nod2-associated phenotype, both sexes were used experimentally (in a gender-matched manner) between the ages of 6–10 weeks. Studies, as well as the method of euthanasia were carried out in accordance with the US Department of Health and Human Services Guide for the Care and Use of Laboratory Animals and were performed under protocols approved by the Institutional Animal Care and Use Committee of the VA Portland Health Care System. To induce euthanasia, CO_2_ was administered from a compressed gas tank for at least 1 min and mice were monitored for absence of heartbeat and respiration. As a secondary physical method of euthanasia, cervical dislocation was performed following CO_2_ administration.

### Induction of EAU

Purified whole bovine IRBP (150 µg) combined with IRBP peptide_1–20_ (300 µg; Anaspec, Fremont, CA) was emulsified in complete Freund’s adjuvant (CFA) containing 2.5 mg/ml heat-killed *Mycobacterium tuberculosis* H37RA (Difco, Detroit MI) as reported^60^ and was injected sub-cutaneously in the morning (9–11 a.m.) of the day of immunization. Mice were also intraperitoneally (i.p.) injected with 0.5 µg Bordetella pertussis toxin (PTX, List Biological Laboratories, Campbell, CA). In the case of adjuvant replacement studies (Supp. Fig. 6), EAU was induced as reported^61^, wherein emulsions were prepared as above, but in lieu of CFA, Incomplete Freund’s adjuvant (IFA) was reconstituted with 2.5 mg/ml heat-killed *Candida albicans* (HKCA; ATCC 10231; Invivogen Carlsbad, CA) or heat-killed *Saccharomyces cerevisiae* (HKSC; ATCC 32119; Invivogen).

### Clinical and histopathological evaluation of disease

Clinical, ocular fundus examinations were performed on anesthetized mice as described^60^. Following pupil dilation with 2.5% phenylephrine hydrochloride (Bausch and Lomb) and 1% tropicamide (Alcon), uveitis was assessed in masked fashion using a previously defined scoring system that ranges from 0 (no disease) to 4 (maximal disease) based on the extent of optic disc inflammation, vasculitis, infiltrative lesions, hemorrhages, and serous retinal detachment. For histopathological scoring of uveitis, at indicated time points, eyes were enucleated and fixed in 10% neutral-buffered formalin, then embedded in paraffin for sectioning. Tissue sections (7 μm) were cut through the pupillary-optic nerve axis at four different depths and three H&E-stained sections from each depth were prepared. Uveitis severity was scored by a masked observer using defined grading criteria ranging from 0 (no disease) to 4 (maximum disease) based on retinal cell damage, retinal detachment, vasculitis, granulomatous-like lesions, hemorrhages, perivascular exudates, and optic disc infiltration^60^. Incidence was calculated based on an animal having achieved a score ≥0.5. For histopathological assessment of pineal gland inflammation, skull caps were dissected at indicated times and fixed in 10% neutral buffered formalin. Fixed pineal glands were then removed from the skull cap, dipped in hematoxylin, and processed for histology. H&E-stained sections (7 µm) were scored for pinealitis by a masked observer using grading criteria based on described methods^62^ that ranged from 0 (no disease) to 3 (severe disease) based on the extent of lymphocytic infiltrates within the pineal gland and adjacent meninges and pinealocyte morphology.

### Electroretinography

To assess retinal function, electroretinograms were recorded on male and female WT and Nod2^−/−^ mice at 8 weeks and again at 12 weeks of age, based on prior methodology^63^. Mice that were dark-adapted overnight were deeply anesthetized with ketamine (100 mg/kg) and xylazine (10 mg/kg), and pupils were dilated with 2.5% phenylephrine and 1% tropicamide eye drops. Gold wire electrodes were placed centrally on the cornea, with a reference electrode in the mouth, and a ground electrode in the tail. To assess rod-driven responses, increasing scotopic stimuli were presented sequentially (−3.7 to 2.6 log scotopic candela (cd)·s/m^2^) in 8 steps using a Colordome Espion electroretinography (ERG) recording system (Diagnosys, Lowell, MA). Following light adaptation for 7 min, a single cone-mediated photopic response was recorded (3.3 log cd·s/m^2^), followed by flicker responses at 1.2 log cd·s/m^2^ over frequencies of 3–30 Hz on a constant white background of 50 cd/m^2^. Responses from both eyes were recorded simultaneously. Scotopic procedures were carried out under red-light conditions and all ERG procedures were conducted with the animal on a 37 °C heating pad.

### Retinal morphology

H&E-stained sections (7 μm) of retinae from naïve WT and Nod2^−/−^ mice (7 weeks age) were imaged at 40× at the Pathology Core Facility, University of Nebraska Medical Center (Omaha, NE) using Panoramic SCAN (Reveal Biosciences, San Diego, CA). Retinal thickness was measured at 200 μm intervals, peripherally from the optic nerve into the inferior and superior hemispheres. Inner and outer nuclear layer thickness measurements were averaged from 6 retinae from 3 separate animals for each genotype. These data were plotted with the center point representing the optic nerve head. All measurements were performed in CaseViewer 2.0 (3DHISTECH Ltd., Budapest, Hungary).

### Evaluation of blood–retinal barrier integrity

BRB integrity was assessed using FITC-dextran based on prior methodology^64^. Briefly, mice were injected intravenously (i.v.) with 100 µl/25 g of 50 mg/ml FITC-dextran (4 kDa; Sigma), and 10 min later mice were euthanized, eyes were harvested into 4% paraformaldehyde, and retinae were dissected. Four radial incisions were made in the retina for mounting in glycerol:PBS (1:1 (v/v)) on a glass slide. Images of retinae were acquired with fluorescence microscopy (Zeiss Apotome) at 5× original magnification. ImageJ with Java 1.8.0 (NIH) was used to quantify fluorescence intensity per region of interest. Retinal autofluorescence was accounted for by normalizing values to those of retinae from saline-injected mice.

### TUNEL assay

A TUNEL (terminal deoxynucleotidyl transferase-mediated dUTP nick-end labeling) assay-based protocol (In Situ Cell Death Detection Kit, Roche) was used to assess extent of DNA fragmentation as an indicator of apoptotic death within the entire retinal thickness. Formalin-fixed, paraffin-embedded eye sections were digested with proteinase K (20 µg/ml, Roche), then incubated with deoxytransferase enzyme, fluorescein-12-dUTP and dATP as per manufacturer’s instructions. SlowFade Gold mounting medium was applied (Life Technologies). Sections treated with 100 U/ml recombinant DNaseI were used as positive control. Epifluorescence images were obtained from 3 different tissue sections per eye (Zeiss Apotome, 5× original magnification). Fluorescence intensity in defined regions of retinal tissue was measured (ImageJ with Java 1.8.0, NIH). The TUNEL score, as a measure of extent of DNA fragmentation, was defined as fluorescence signal intensity per # of DAPI^+^ cells/mm^2^ retina.

### Adoptive T cell-transfer model

Adoptive transfer studies were carried out based on prior methods^65^. Splenocytes were isolated 14 days post-immunization from IRBP-immunized WT or Nod2^−/−^ donor mice and pooled (*n* = 14–16 mice/group). Splenocytes that were cultured in vitro with 20 µg/ml IRBP_1–20_ for 3 days at 37 °C and 5% CO_2_ were then enriched for live cells over Lympholyte-M columns and negatively selected for CD4^+^ T cells (EasySep™, STEMCELL Technologies, Inc.), resulting in >99% purity of CD4^+^ T cells. Naïve syngeneic WT recipients were i.v. injected with the purified CD4^+^ T cells (2 × 10^7^) and i.p. injected with PTX (0.5 μg), then monitored for uveitis development.

### EAU induction in reconstituted lymphopenic Rag1^−/−^ mice

CD4^+^ T cells were isolated (to >99% purity) from spleens of naïve WT or Nod2^−/−^ donor mice using immunomagnetic negative selection (CD4^+^ Negative Selection Kit #19852, StemCell Technologies) and i.v. injected (3.5 × 10^7^ cells) into naïve hosts: Rag1^−/−^ or Nod2^−/−^Rag1^−/−^ mice. Levels of T cell reconstitution were verified to be comparable (both numerically and proportionally, ~40% of live blood cells) at time of immunization, i.e., 1 day post-transfer. Body weight was monitored from the time of cell transfer to termination of study (i.e., 4–5 weeks post-transfer) and there was no significant weight loss measured within this time-frame for either genotype. For thymocyte transfers, thymi were harvested from naïve WT, Nod2^−/−^, or Rip2^−/−^ donor mice, dissociated through a 70 µm filter, and i.v. injected (10^8^ cells in 150 µl) into congenic Rag1^−/−^ recipients. Levels of T cell reconstitution were verified to be comparable (numerically and proportionally ~32–35% of live blood cells) at the time of immunization (i.e., 8 days post-transfer).

### Uveitis induction by transfer of R161M-Tg T cells

CD4^+^ T cells were negatively-selected (EasySep) from spleens of naïve R161M or Nod2^−/−^R161M mice and i.p. injected (10^6^ cells) into Rag2^−/−^ (B10.RIII) recipients, who were then monitored out to 7 days for onset of uveitis; after which the uveitis became too severe to accurately quantify.

### Generation of bone marrow chimeric mice

WT or Nod2^−/−^ recipient mice (6 weeks of age) were lethally irradiated with a total of 1100 cGy (delivered in two 550 cGy doses administered 3 h apart) (X-RAD 320, Precision X-ray, North Branford, CT), then transplanted with BM cells (4 × 10^6^ cells) flushed and filtered (40 µm) from femurs of WT or Nod2^−/−^ donor mice. Following transplantation, mice were administered ciprofloxacin (0.2 mg/ml) in drinking water that was refreshed every 3 days for 3 weeks, after which mice were assessed for hydration status by weighing and skin/fur evaluation. Chimeric mice achieved >95% donor engraftment 8 weeks after transplantation, as confirmed on the basis of congenic CD45.1/CD45.2 antigen expression (using CD45.1-expressing B6.SJL-Ptprc^a^ Pepc^b^/BoyJ (Jackson Laboratory stock #002014). Chimeric mice (CD45.2 C57BL/6J) were subsequently induced for EAU by standard protocol as above.

### Cohousing and broad spectrum antibiotics

WT and Nod2^−/−^ mice were cohoused for 4 weeks (starting at 3–4 weeks of age, at the time of weaning) at a 1:1 ratio to equally expose each genotype to a composite of fecal and bedding material prior to experiment. Mice remained cohoused throughout the duration of the study (i.e., out to 3 weeks post-immunization). For broad-spectrum antibiotic treatment, mice were orally administered ampicillin (1 g/l), metronidazole (1 g/L), neomycin (1 g/L), vancomycin (500 mg/L) in their drinking water (as described^20^) for 3 weeks prior to induction of EAU.

### In vivo CD4^+^ T cell depletion

Rat-anti-mouse CD4 mAb (clone GK1.5, IgG2b) was generated from hybridoma cultures (GK1.5; ATCC TIB-207; Manassas, VA), purified using HiTrap Protein-G columns (ThermoFisher Scientific Pierce), dialyzed, and sterilized by membrane filtration. Mice were administered i.p. injections of anti-CD4 mAb (100 µg/injection) or rat IgG2b isotype antibody (clone 141945; R&D Systems) on days −1, 0, 1, 6, 11, 16 relative to immunization (day 0). In our hands, we achieved >95% depletion of circulating T cells (as confirmed by flow cytometry).

### In vivo neutralization of IL-17 and IFNγ

For neutralization of IL-17 or IFNγ, mice were administered i.p. injections (100 µg) of anti-mouse IL-17A/F mAb (clone 50104; R&D systems), anti-mouse IFNγ mAb (clone 37895, R&D) or anti-mouse IgG2a mAb (clone 20102; R&D) on days, −1, 0, 2, 5, 9, 13 relative to immunization (d0), as established^28,66–68^.

### Flow cytometry on eyes and pineal glands

Eyes were enucleated and the intraocular lens removed. Pineal glands were dissected as described above. Single-cell suspensions were prepared from eyes (pooled *n* = 6 mice/genotype/condition) or pineal glands (pooled *n* = 5–6 pineal glands/genotype/condition) by collagenase-digestion (1 mg/ml Clostridium histolyticum Collagenase D, Roche) for 40 min at 37 °C and sequential filtration through a 70-µm and 40-µm strainer as described^60^. Cells were stained with live/dead fluorescent dye 1:200 dilution (Viability Dye eFluor® 506; eBioscience) and incubated with Fc block (3 µg/ml, BD Pharmingen) for 30 min on ice. Cells were then incubated with fluorescently labeled anti-mouse antibodies (BD Biosciences) against: CD45 1:50 dilution (30-F11), Thy1.2 1:50 dilution (53-2.1), CD3 1:50 dilution (145-2C11), CD4 1:50 dilution (RM4-5), CD8α 1:50 dilution (53-6.7), CD69 1:1000 dilution (H1.2F3), CD44 1:50 dilution (IM7), CD62L 1:50 dilution (MEL-14), CD11b 1:50 dilution (M1/70), Gr-1 1:50 dilution (RB6-8C5), IL-23R 1:50 dilution (O78^−^1208), B220 1:50 dilution (RA3-6B2), Ly6G 1:50 dilution (1A8), F4/80 1:50 dilution (BM3, BioLegend), and CD11c 1:1000 dilution (HL3). Following fixation (4% paraformaldehyde), cells were analyzed on an LSRFortessa (BD Biosciences). Dead cells were excluded based on viability dye staining so that only live cell events were collected. FlowJo 9.0 (Becton, Dickinson, and Company, Franklin Lakes, NJ, USA) was used for analysis, allowing compensations for spectral overlaps within each sample. Gating used defined criteria based on control samples stained with corresponding isotype control antibodies (IC Ab) and equal number of total live events were collected. Live cells were gated from forward and side scatter plots in identical fashion for each group, after which frequencies and numbers of leukocyte subpopulations were determined from CD45^+^ gated cells. T cells were identified as being positive for Thy1.2 or CD3, positive for either CD4 or CD8, and negative for B220.

### Proliferation assay

IRBP-specific lymphocyte proliferation was measured by [^3^H]-thymidine incorporation based on prior methods^60^. Cells (5 × 10^5^/ml) were seeded into 96-well plates and stimulated with 20 μg/ml IRBP_1–20_ peptide for 72 h. The wells were pulsed for the final 18 h with 0.5 µCi per well [3H]-thymidine and uptake was determined using liquid scintillation spectrometry (Wallac model, WinSpectral).

### Antigen-T cell stimulation for single or multiplex ELISA

To measure cytokine production of IRBP-responsive CD4^+^ T cells, single cell suspensions from spleens were prepared from immunized mice, and erythrocytes were lysed using red blood cell lysis buffer (Sigma) as described^60^. Cells (1–2 × 10^6^ cells/well) were seeded in 96-well V-bottom polypropylene plates (Corning Inc., Corning, NY) in HL-1 medium (Lonza, Basel, Switzerland) supplemented with 5% FBS, 10 μg/ml gentamycin (Sigma, St Louis, MO), 10 mM HEPES (BioWhittaker, Walkersville, MD), 1 mM sodium pyruvate (BioWhittaker), and nonessential amino acids (BioWhittaker) and stimulated with 20 μg/ml IRBP_1–20_ peptide. After 18 h, concentrations of cytokines (IL-10, IL-12p40, IP-10, KC (aka Cxcl1), TNFα, IL-2, IL-6, IL-17A/F, IFNγ) were measured using a Luminex® multiplex assay (Millipore, Billerica, MA, USA) on Model L100IS (Luminex, Austin, TX, USA) and analyzed with BeadView™ (Upstate Cell Signaling Solutions, Lake Placid, NY, USA) as described^60^ (Fig. 3). Sandwich ELISAs for IL-17A or IFNγ were carried out as per manufacturer’s instructions (DuoSet, R&D). For stimulation of R161M-Tg cells, splenocytes from naïve R161M mice were processed as above, but were seeded in serum-free medium (X-Vivo 15, Lonza) in the presence of MDP (10 µg/ml), IRBP peptide_161–180_ (20 µg/ml), or PMA/ionomycin (20 ng/ml PMA (LC Labs) + 1 µg/ml ionomycin (LC Labs)). Cell supernatant was collected after 72 h stimulation for evaluation of IL-17 by ELISA as per manufacturer’s instruction (DuoSet, R&D).

### T cell stimulation and intracellular cytokine staining

Single cell suspensions from spleens or eyes (pooled *n* = 6 mice/genotype/group) were prepared and erythrocytes were lysed using red blood cell lysis buffer (Sigma). Cells (1–2 × 10^6^ cells/well) were seeded in 96-well V-bottom polypropylene plates (Corning Inc., Corning, NY) in serum-free HL-1 medium (Lonza, Basel, Switzerland) and stimulated in the presence of Brefeldin A (1 µg/ml; BD GolgiPlug™, BD Biosciences) with either 20 μg/ml IRBP_1–20_ peptide (eBioscience) for 18 h or with 20 ng/ml PMA + 200 ng/ml ionomycin for 6 h. Cells were blocked with mAb to FcγRIII/I (2.4G2, BD) and subsequently incubated with LIVE/DEAD™ Aqua 1:200 dilution (Life Technologies) along with mAb to: CD3 1:1000 dilution (145-2C11, BD Bioscience), Thy1.2 1:50 dilution (53-2.1, BioLegend), CD8α 1:1000 dilution (53-6.7, eBiosciences) and CD4 1:50 dilution (RM4^−^5, BD), followed by fixation and permeabilization (BD Cytofix/Cytoperm^TM^ Fixation/Permeabilization solution Kit (BD Biosciences). Cells were then incubated with fluorescently-labeled antibodies (BD Bioscience) specific to: IL^−^17A 1:50 dilution (TC11-18H10, BD Pharmingen), TNFα 1:1000 dilution (MP6-XT22, BD Horizon), IFNγ 1:1000 dilution (XMG1.2, BD Pharmingen), GM-CSF 1:50 dilution (MP1-22E9, BioLegend), IL-22 1:50 dilution (IL-22JOP, eBioscience), IL-2 1:1000 dilution (JES6/5H4, BD Biosciences), and Foxp3 1:50 dilution (FJK-16s, eBioscience). Flow cytometry was performed on BD LSRFortessa™ and then analyzed using FlowJo 9.0 (Becton, Dickinson, and Company).

### Multiplex-quantitative real-time-PCR on CD4^+^ T cells

At 18 h following IRBP peptide_1–20_ stimulation (as described in antigen-stimulation assays in splenocytes above), live mouse cells from adjuvant or IRBP-immunized mice were enriched over Lympholyte-M columns, followed by immunomagnetic positive selection for CD4^+^ T cells (EasySep CD4^+^ Positive Selection Kit II (StemCell Technologies), and subsequent FACS-sorted for live (negative for propidium iodide, ThermoFisher) B220^−^Thy1.2^+^CD8^−^CD4^+^ T cells (BD Influx Cell Sorter) that resulted in >99% CD4^+^ T cell purity. Total RNA was extracted as described^60^ on 10^6^ cells (RNeasy kit, Qiagen). cDNA was then synthesized (Reverse Transcription Kit; Applied Biosystems), added to RT^2^ SYBER Green ROX qPCR Master Mix (Qiagen), and multiplex-transcript analysis of Th17-associated genes was performed (as in Fig. 3b) using quantitative real-time PCR (RT^2^Profiler^TM^ PCR Gene Expression Assay kit, SABiosciences) and Applied Biosystems PRISM Sequence Detection system. Controls were performed to confirm the lack of contaminating genomic DNA. Levels of target gene expression were determined by standard comparative ΔΔCt method and normalized to the average of 5 housekeeping genes within the same sample (Gusb, Hprt, Hsp90ab1, Gapdh, Actb), all of which were expressed at similar levels. For Fig. 3b, array results were combined from 2 studies (*n* = 6 mice pooled/group) and are expressed as fold induction relative to IRBP-stimulated CD4^+^ T cells from adjuvant-injected animals.

### Nod2 transcriptional analysis

Quantitative real-time PCR for Nod2 and β-actin (Actb) products was carried out on total RNA extracted from equal numbers of purified CD4^+^ T cells (>99% CD4^+^ T cell purity) or BM-derived macrophages generated as detailed^56^, or on dissected ocular tissues (cornea, iris/ciliary body, retina, retinal pigment epithelium (RPE)/choroid that were pooled from 6 transcardial, saline-perfused mice; Supp. Fig. 3). As described^56^, qPCR was performed with iQ Syber-Green Supermix (BioRad, Hercules, CA) using forward and reverse primers for Nod2, 5′-AAGAGCAACACCTCCCTGAA-3′ and 5′-AGCATCAGTGCTAGGGCTTC-3′; for Actb (1760-1761) 5’–TTCTACAATGAGCTGCGTGTG–3′ and 5′–GGGGTGTTGAAGGTCTCAAA–3′. Controls were performed to confirm the lack of contaminating genomic DNA. Levels of target gene expression were determined by the standard comparative ΔΔCt method and normalized to Actb. Results are mRNA transcript expression normalized to Nod2 mRNA transcript within naïve WT-spleen RNA, and are representative of 3 independently performed experiments (each sample contained RNA pooled from 6 mice resulting in 18 total mice/genotype analyzed).

### In vitro autologous co−cultures

APCs were enriched from spleens of naïve WT or Nod2^−/−^ mice (pooled *n* = 3 mice/genotype) that were depleted of T cells (using EasySep Positive selection kit II for CD90.2/Thy1.2 cells; StemCell Technologies), and preparations were confirmed by flow cytometry to be <3% T cells. APCs were seeded (1.2 × 10^6^ cells/well) in round-bottom 96-well plates and stimulated for 2 h with 20 μg/ml IRBP_1–20_ prior to the addition of purified CD4^+^ T cells (3 × 10^5^ cells/well, T cell:APC ratio of 1:4). CD4^+^ T cells (confirmed to be >99% pure) were isolated by negative selection (CD4^+^ T cell isolation Kit; StemCell Technologies) from spleens of immunized WT or Nod2^−/−^ mice (pooled *n* = 3 mice/genotype; 14 days post-immunization). Culture supernatants were collected after 18 h co-culture, and levels of IL-17 and IFNγ were analyzed by sandwich ELISAs (DuoSet, R&D).

### In vitro Th17 differentiation of naïve CD4^+^ T cells

Naïve CD4^+^ T cells (>99% purity, CD62L^hi^CD44^−^) were isolated from spleens of WT or Nod2^−/−^ mice by negative selection (StemCell). Cells were then differentiated in vitro according to manufacturer’s instruction (CellXVivo mouse Th17 cell differentiation kit, R&D systems): 1.25 × 10^5^ naïve CD4^+^ T cells were incubated in serum-free X-Vivo 15 medium (Lonza) containing penicillin/streptomycin and in the presence of CD3 mAb, CD28 mAb and Th17-polarizing cytokines as provided by the kit. After 5 days of culture, CD4^+^ T cells were stimulated with PMA/ionomycin in the presence of brefeldin A and the frequency of IL-17^+^ CD4^+^ T cells was quantified by flow cytometry (as above).

### In vitro stimulation of memory CD4^+^ T cells with CD3/CD28

Memory CD4^+^ T cells were isolated (to >99% purity, CD62L^−^CD44^+^) from spleens of naïve WT or Nod2^−/−^ mice (StemCell 19767). Purified cells were suspended in serum-free X-Vivo 15 medium (Lonza) containing penicillin/streptomycin, CD28 (37.51, 2 µg/ml), and brefeldin A and seeded at 1.25 × 10^5^ cells/ well of a 96-well plate that had been pre-coated with CD3e mAb (145-2C11, 10 µg/ml). After 5 d, T cells were stimulated with PMA/ionomycin in the presence of brefeldin A for 4 h followed by intracellular cytokine staining and flow analysis performed as above. In the case of Fig. 7f, g, purified memory T cells were stimulated overnight (16 h) and cells harvested for RNA isolation (RNeasy Mini Kit) and analyzed for transcriptional response by multiplex PCR array (as detailed above, *n* = 3 mice/genotype/group). The mRNA for each sample was then converted into cDNA (RT^2^ First Strand Kit, Qiagen), which was added to the RT^2^ SYBER Green ROX qPCR Master mix (Qiagen) and run on a 96-well RT^2^ Profiler PCR Array Mouse Inflammatory Response and Autoimmunity plate (Qiagen) using QuantStudio 3.0 (Applied Biosystems), and analyzed as detailed below. Controls were performed to confirm the lack of contaminating genomic DNA. Levels of target gene expression were determined by standard comparative ΔΔCt method and normalized to the average of 5 housekeeping genes within the same sample (Gusb, Hprt, Hsp90ab1, Gapdh, Actb), all of which were expressed at similar levels. Array results are expressed as fold induction relative to unstimulated CD4^+^ T cells of naïve mice.

### Stimulation of human T cells

All uses of human material have been approved by the Institutional Review Boards of the University of Minnesota and Oregon Health & Science University. All recruited volunteers provided written informed consent. Human CD4^+^ T cell stimulations were carried out on viable frozen PBMCs from Blau syndrome patients vs. healthy, gender-matched controls. The two related patients with Blau syndrome (confirmed heterozygous CARD15c.1147G->A mutation, leading to pGlu383Lys mutation) included a mother (Blau-2, 48−year-old female) and son (Blau-1, 17-year-old male), who had onset of disease at ages 15 and 9, respectively. Blau-1 had been previously treated with methotrexate and was on adalimumab to control dermatitis and arthritis at the time of blood draw. Blau-2 had been on adalimumab and infliximab in the past and was on abatacept, methotrexate, prednisone (5–15 mg daily), and alendronate to control arthritis, uveitis, and nodular skin lesions with granulomatous inflammation (confirmed by biopsy) at the time of blood draw. Peripheral blood was collected into BD Vacutainer ® CPT Mononuclear Cell Preparation tubes (Sodium Citrate) and PBMCs were isolated per manufacturer’s instructions (BD Bioscience) within 24 h of collection. PBMCs were washed twice in sterile 1× PBS and cryopreserved in freezing medium until use. In Fig. 7h, PMBCs were incubated in round-bottom 96-well plates (10^6^cells/well) in serum-free X-Vivo 15 media for 5 days, and stimulated with PMA/ionomycin for the last 4 h. In Fig. 7i, PBMCs were stimulated for 5 days in serum-free X-Vivo 15 media in the presence of plate-bound anti-CD3 (10 μg/ml; clone HIT3a; BD Biosciences) and anti-CD28 (5 μg/ml; clone CD28.2; BD Biosciences). Cells were then stained with LIVEDEAD Aqua, and antibodies to CD3 1:1000 dilution (UCHT1, BD Biosciences), CD8 1:50 dilution (SK1, BD Biosciences), CD4 1:50 dilution (SK3, BD Biosciences), CD19 1:1000 dilution (HIB19), IL^−^17A 1:50 dilution (eBio64DEC17, eBioscience), or CCR7 1:50 dilution (G043H7, BioLegend).

### Statistical analysis

Data are presented as mean ± SEM (with dots representing individual values), box-whisker plots showing median with 25th–75th percentile and min–max range or floating bars showing median with min–max range. Complex data sets were compared by ANOVA for multiple comparisons. Statistical differences between individual groups were calculated using the two-tailed Mann–Whitney U test or the unpaired, two-tailed Student’s *t*-test using Prism 8.0 (GraphPad Software Inc.). For the in vitro studies with human samples, statistical differences between 2 groups was determined using an unpaired, two-tailed Student’s *t*-test which has been validated for use when comparator groups have *n* values between 2 and 5^69^. Values of *p* < 0.05 were considered to be statistically significant.

### Reporting summary

Further information on research design is available in the Nature Research Reporting Summary linked to this article.

## Supplementary information

Supplementary Information

Reporting Summary

## Data Availability

All other data are included in the supplemental information or will be made available from the authors upon reasonable requests. Source data are provided with this paper that contains raw data presented in Figs. 1–7 and in Supplementary Figs. 1–6. Source data are provided with this paper.

## Source data

Source Data
