# Supplementary material for: T cell-intrinsic role for Nod2 in protection against Th17-mediated uveitis
Source: Nat Commun. 2020 Oct 26;11:5406. doi: 10.1038/s41467-020-18961-0 (PMC7589501; doi:10.1038/s41467-020-18961-0)
Supplement: Supplementary file 1 — Supplementary Information [file 41467_2020_18961_MOESM1_ESM.pdf]

Supplementary information

T cell-intrinsic role for Nod2 in protection against Th17-mediated uveitis

Ruth J. Napier*, Ellen J. Lee*, Michael P. Davey, Emily E. Vance, João M. Furtado, Paige E. Snow, Kimberly A. Samson, Sydney J. Lashley, Brieanna R. Brown, Reiko Horai,Mary J. Mattapallil, Biying Xu, Michelle C. Callegan, Luke S. Uebelhoer, Christina L. Lancioni, Richard K. Vehe, Bryce A. Binstadt, Justine R. Smith, Rachel R. Caspi, Holly L. Rosenzweig

*These authors contributed equally

Corresponding author: Holly L. Rosenzweig

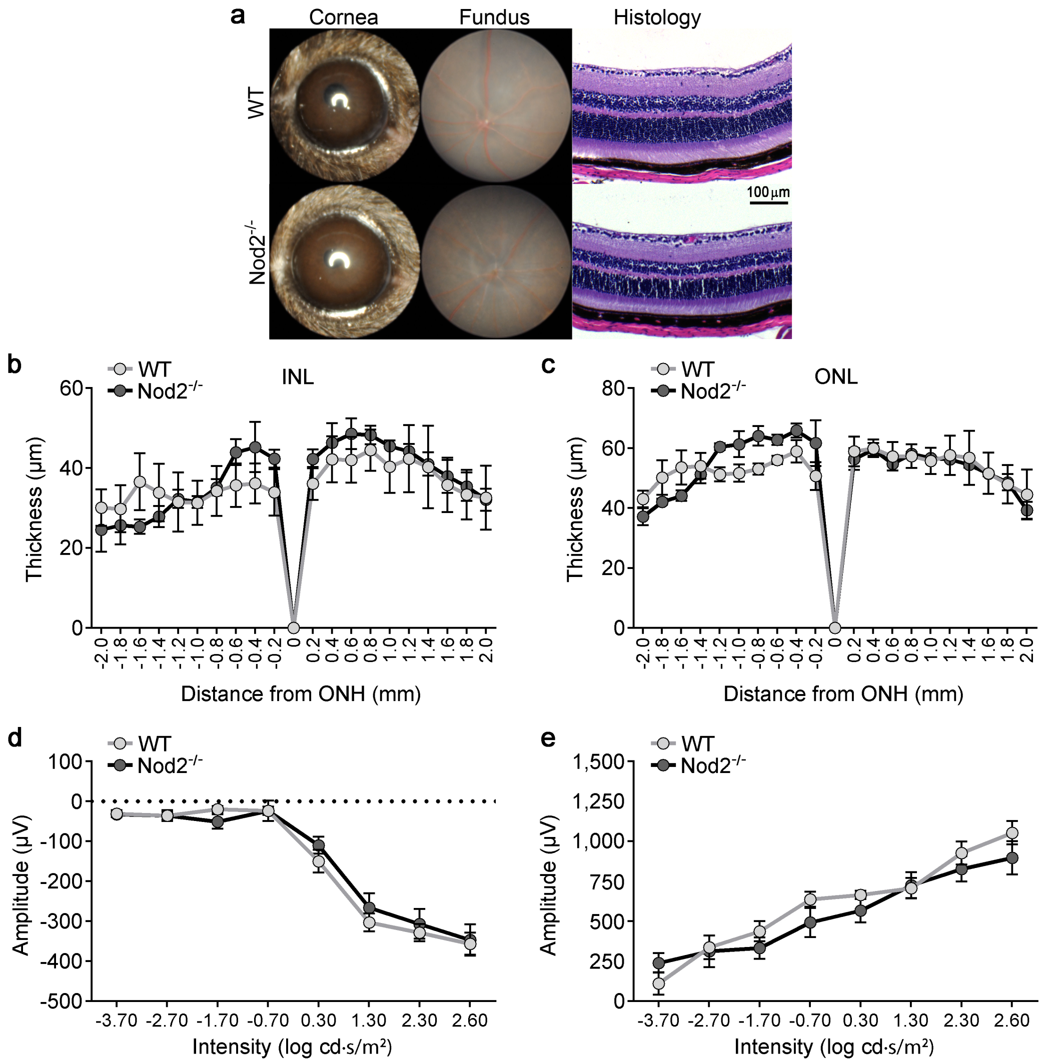

Supplemental Figure 1. Anatomical and functional evaluation of eyes from Nod2-/- mice. (a) Eyes of naive WT and Nod2-/- mice were examined under magnification with a dissecting microscope (left), by fundoscopy (center), and by histology (right). The thickness of: (b) inner nuclear layer (INL) and (c) outer nuclear layer (ONL) of retinas of 8 week old WT and Nod2-/- mice was assessed. Measurements were made from H&E-stained sections (6-8 sections obtained per mouse, n = 3 mice/genotype and are representative of 2 experiments, p=NS). (d, e) Electroretinography of 8 wk old WT and Nod2-/- mice was performed to evaluate retinal function. Scotopic A-wave (d) and scotopic B-wave (e) amplitudes were used to assess functional cell responses dominated by photoreceptor and bipolar cell activity, respectively. Recordings were made from 6 mice/genotype and are representative of 2 independent experiments (p=NS). Data are mean $\pm$ SEM. Statistical differences between groups were calculated using two-tailed Mann-Whitney U test (b,c) and unpaired Student's t-test (d, e). Source data are provided as a Source Data file.

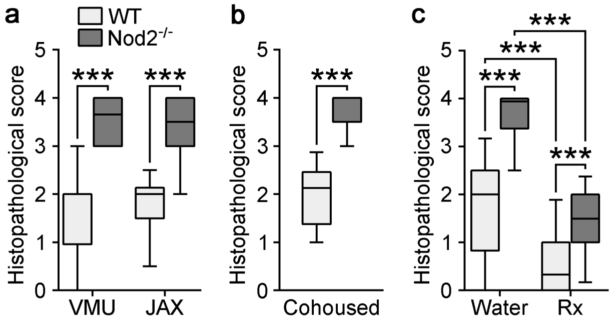

Supplemental Figure 2. Examination of microbiota influence on uveitis. (a) To evaluate colony-associated differences, WT and Nod2$^{-/-}$ mice, either bred in the VA Medical Unit (VMU) or newly arrived (within one day of experiment) from The Jackson Laboratory (JAX), were immunized with IRBP and evaluated by histopathology 20 days post-immunization. Data are combined from 2 independent experiments for n =10 (JAX-WT and VMU-Nod2$^{-/-}$), 11 (JAX-Nod2$^{-/-}$) and 18 (VMU-WT) mice. ***p<0.0001 (VMU comparison) and ***p = 0.0005 (JAX comparison). (b) To evaluate strain-specific microbiota, EAU was induced in WT and Nod2$^{-/-}$ mice that were cohoused from the time of weaning (i.e. 4 weeks prior to immunization) and for the duration of the experiment. Uveitis was evaluated by histopathology 20 days post-immunization. Data are combined from 2 independent experiments where n =16 mice/group. ***p<0.0001. (c) Mice were orally administered broad-spectrum antibiotics in their drinking water for 3 weeks prior to induction of EAU and uveitis was evaluated 20 days post-immunization by histopathology. Data are combined from 2 independent experiments where n = 23 (WT-water), 21 (WT-Rx), 14 (Nod2$^{-/-}$ water), 15 (Nod2$^{-/-}$ Rx) mice. ***p<0.0001 (WT water vs. WT Rx, Nod2$^{-/-}$ water vs. Nod2$^{-/-}$ Rx, WT water vs. Nod2$^{-/-}$ water) and ***p = 0.0002 (WT Rx vs. Nod2$^{-/-}$ Rx). Data are box-whisker plots showing median, 25-75th percentile, and min – max range. Statistical differences between groups were calculated using the two-tailed Mann-Whitney U test. Source data are provided as a Source Data file.

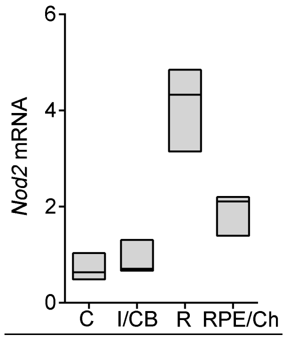

Supplemental Figure 3. ***Nod2* expression in the eye.** qPCR was used to evaluate mRNA expression of *Nod2* in distinct ocular tissues of naïve C57BL/6J mice, which are expressed as relative to mRNA expression of *Nod2* in spleen. Data are combined from 3 independent experiments (for each, dissected ocular tissues were pooled from n = 6 mice for RNA purification). Data are presented as floating bars with min – max range. C; cornea; I/CB, iris/ciliary body; R, retina; RPE/Ch, retinal pigment epithelium/choroid. Source data are provided as a Source Data file.

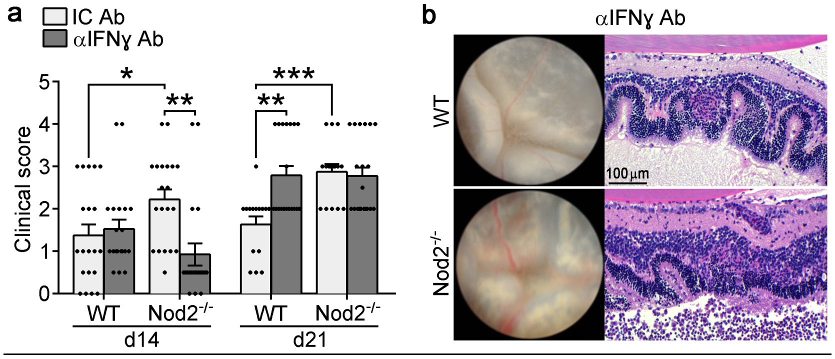

Supplemental Figure 4. The role of IFNγ in uveitis in WT and Nod2$^{-/-}$ mice. The effect of IFNγ neutralization via administration of anti-IFNγ Ab or isotype control (IC) Ab on uveitis in WT and Nod2$^{-/-}$ mice was assessed. (a) Clinical uveitis as evaluated by fundoscopy was scored repeatedly. Data are combined from 3 independent experiments where on day 14, n = 20 for all conditions. For day 21, n = 15 (WT IC), 19 (WT anti-IFNγ), 16 (Nod2$^{-/-}$ IC), and 20 (Nod2$^{-/-}$ anti-IFNγ). For 14 days; **p=0.0007 for Nod2$^{-/-}$ IC vs. Nod2$^{-/-}$ anti-IFNγ, *p=0.0189 for WT IC vs. Nod2$^{-/-}$ IC. For 21 days; **p = 0.0006 for WT IC vs. WT anti-IFNγ, ***p<0.0001 for WT IC vs. Nod2$^{-/-}$ IC. (b) Representative images of fundi and H&E-stained retinal sections 21 days post-immunization. Data are mean ± SEM with dots representing individual values. Statistical differences between groups were calculated by unpaired Student's t-test. Source data are provided as a Source Data file.

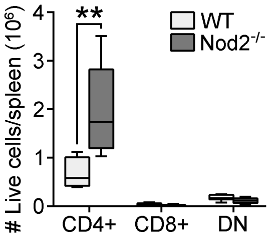

Supplemental Figure 5. Increased expansion of Nod2$^{-/-}$ CD4^{+} T cells in IRBP-immunized lymphopenic hosts. Splenocytes harvested from IRBP-immunized Rag1$^{-/-}$ mice that had received either WT or Nod2$^{-/-}$ CD4^{+} T cells were analyzed by flow cytometry for total numbers of indicated T cell subsets. Data are combined from 2 independent experiments where n = 6 (WT CD4^{+}), 9 (Nod2$^{-/-}$ CD4^{+}, Nod2$^{-/-}$ CD8^{+}, Nod2$^{-/-}$ CD4^{-}CD8^{-} "DN"), 8 (WT CD8^{+}), 7 (WT DN) mice, **p=0.0008. Data are box-whisker plots with medians, 25-75th percentile, and min-max range, and were compared using the two-tailed Mann-Whitney U test. Source data are provided as a Source Data file.

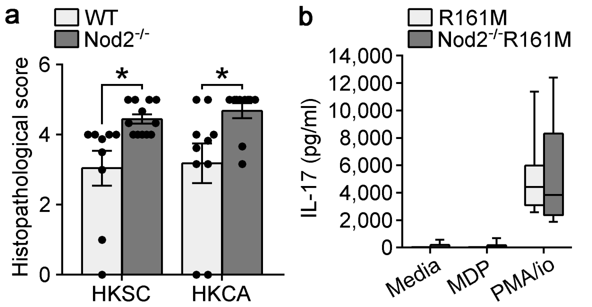

Supplemental Figure 6. Nod2-mediated protection against uveitis is independent of CFA-containing immunization or MDP-stimulation. To evaluate a CFA-independent means of EAU induction, fungal-triggered uveitis was induced by immunization with IRBP emulsified in IFA containing heat-killed *S. cerevisiae* (HKSC) or heat-killed *C. albicans* (HKCA). Uveitis was evaluated by histopathology in WT and Nod2$^{-/-}$ mice 20 days post-immunization using an expanded scale ranging 0 to 5. Data are combined from 2 independent experiments. For HKSC; n = 9 (WT) and 12 (Nod2$^{-/-}$) and for HKCA; n = 10 mice/group. *p = 0.0062 (HKSC) and *p = 0.0151 (HKCA). (b) Splenocytes from naïve R161M or Nod2$^{-/-}$R161M mice were stimulated *in vitro* with MDP (10 μg/ml), versus PMA/ionomycin as a positive control. Culture supernatants were collected 72 hours post-stimulation and IL-17 production was evaluated by ELISA. Data is representative of 2 independent experiments where n = 5 mice/group. P = NS between WT and Nod2$^{-/-}$ responses. Data are mean $\pm$ SEM with dots representing individual values (a), or box-whisker plots with median, 25-75th percentile, and min – max range (b). Statistical differences between groups were calculated using unpaired Student's t-test (a) or Mann-Whitney U test (b). Source data are provided as a Source Data file.
