# Supplementary material for: T cell-intrinsic role for Nod2 in protection against Th17-mediated uveitis
Source: Nat Commun. 2020 Oct 26;11:5406. doi: 10.1038/s41467-020-18961-0 (PMC7589501; doi:10.1038/s41467-020-18961-0)
Supplement: Supplementary file 2 — Reporting Summary [file 41467_2020_18961_MOESM2_ESM.pdf]

nature research

Corresponding author(s): Holly Rosenzweig

Last updated by author(s): Sep 10, 2020

Reporting Summary

Nature Research wishes to improve the reproducibility of the work that we publish. This form provides structure for consistency and transparency in reporting. For further information on Nature Research policies, see our Editorial Policies and the Editorial Policy Checklist.

Statistics

For all statistical analyses, confirm that the following items are present in the figure legend, table legend, main text, or Methods section.

n/a	Confirmed	
☐	☒	The exact sample size (n) for each experimental group/condition, given as a discrete number and unit of measurement
☐	☒	A statement on whether measurements were taken from distinct samples or whether the same sample was measured repeatedly
☐	☒	The statistical test(s) used AND whether they are one- or two-sided<br>*Only common tests should be described solely by name; describe more complex techniques in the Methods section.*
☐	☒	A description of all covariates tested
☐	☒	A description of any assumptions or corrections, such as tests of normality and adjustment for multiple comparisons
☐	☒	A full description of the statistical parameters including central tendency (e.g. means) or other basic estimates (e.g. regression coefficient) AND variation (e.g. standard deviation) or associated estimates of uncertainty (e.g. confidence intervals)
☐	☒	For null hypothesis testing, the test statistic (e.g. F, t, r) with confidence intervals, effect sizes, degrees of freedom and P value noted<br>*Give P values as exact values whenever suitable.*
☒	☐	For Bayesian analysis, information on the choice of priors and Markov chain Monte Carlo settings
☒	☐	For hierarchical and complex designs, identification of the appropriate level for tests and full reporting of outcomes
☒	☐	Estimates of effect sizes (e.g. Cohen's d, Pearson's r), indicating how they were calculated

Our web collection on statistics for biologists contains articles on many of the points above.

Software and code

Policy information about availability of computer code

Data collection	Colordome Espion electroretinography (ERG) recording system (Diagnosys), Panoramic SCAN (Reveal Biosciences), LSRFortessa (BD Biosciences), Applied Biosysystems PRISM Sequence Detection system (SABiosciences),
Data analysis	CaseViewer 2.0 (3DHISTECH Ltd.), ImageJ with Java 8.0 (NIH), FlowJo 10.0 (Becton Dickinson & Co.), Prism 8.0 (GraphPad).

For manuscripts utilizing custom algorithms or software that are central to the research but not yet described in published literature, software must be made available to editors and reviewers. We strongly encourage code deposition in a community repository (e.g. GitHub). See the Nature Research guidelines for submitting code & software for further information.

Data

Policy information about availability of data

All manuscripts must include a data availability statement. This statement should provide the following information, where applicable:
- Accession codes, unique identifiers, or web links for publicly available datasets
- A list of figures that have associated raw data
- A description of any restrictions on data availability

All other data are included in the supplementary information or will be made available from the authors upon reasonable request.

Field-specific reporting

Life sciences study design

All studies must disclose on these points even when the disclosure is negative.

Sample size	A power analysis determined that for in vivo studies, a group size of n=6 gives us statistical power to detect a mean different of 1.33 times the standard deviation, especially considering the variation that is inherently involved in the experimental mouse model of uveitis. Due to inter-animal variability in certain experiments and/or modest treatment effects, then we combined data from 2 or more independently performed studies in order to have a great statistical power.
Data exclusions	Data was rarely excluded. In some odd cases, an individual data point was excluded based on the statistical definition of it being an outlier (which was defined as 2-times the standard deviation above or below the mean). There were also situations where a data point was excluded as the animal became sick and was humanely euthanized and removed from the study.
Replication	All experiments were replicated independently on a separate group of mice (the number of replicated experiments are defined within the figure legends).
Randomization	Mice were randomly assigned to treatment groups based on their genotype.
Blinding	Uveitis (both clinically and histologically) was evaluated in a masked fashion as indicated in the Methods section. Since all mice were assigned a code at the time of immunization for the purpose of repeated clinical evaluation, the collection of samples for in vitro experimentation were also often carried out in a masked fashion.

Reporting for specific materials, systems and methods

We require information from authors about some types of materials, experimental systems and methods used in many studies. Here, indicate whether each material, system or method listed is relevant to your study. If you are not sure if a list item applies to your research, read the appropriate section before selecting a response.

Materials & experimental systems

n/a	Involved in the study	
☐	☒	Antibodies
☒	☐	Eukaryotic cell lines
☒	☐	Palaeontology and archaeology
☐	☒	Animals and other organisms
☐	☒	Human research participants
☒	☐	Clinical data
☒	☐	Dual use research of concern

Methods

n/a	Involved in the study	
☒	☐	ChIP-seq
☐	☒	Flow cytometry
☒	☐	MRI-based neuroimaging

Antibodies

Antibodies used	The following anti-mouse antibodies were used from BD Biosciences (or as indicated): CD45 1:50 dilution (30-F11), Thy1.2 1:50 dilution (53-2.1), CD3 1:50 dilution (145-2C11), CD4 1:50 dilution (RM4-5), CD8α 1:50 dilution (53-6.7), CD69 1:1000 dilution (H1.2F3), CD44 1:50 dilution (IM7), CD62L 1:50 dilution (MEL-14), CD11b 1:50 dilution (M1/70), IL-23R 1:50 dilution (O78-1208), B220 1:50 dilution (RA3-6B2), Ly6G 1:50 dilution (1A8), F4/80 1:50 dilution (BM3, BioLegend), and CD11c 1:1000 dilution (HL3). IL-17A 1:50 dilution (TC11-18H10, BD Pharmingen), TNF 1:1000 dilution (MP6-XT22, BD Horizon), IFNγ 1:1000 dilution (XMG1.2, BD Pharmingen), GM-CSF 1:50 dilution (MP1-22E9, BioLegend), IL-22 1:50 dilution (IL-22JOP, eBioscience), IL-2 1:1000 dilution (JES6/5H4, BD Biosciences), and Foxp3 1:50 dilution (FJK-16s, eBioscience).<br><br>The following anti-human antibodies were used: CD3 1:1000 dilution (UCHT1, BD Biosciences), CD8 1:50 dilution (SK1, BD Biosciences), CD4 1:50 dilution (SK3, BD Biosciences), CD19 1:1000 dilution (HIB19) production of IL-17A 1:50 dilution (eBio64DEC17, eBioscience) or CCR7 1:50 dilution (G043H7, BioLegend).
Validation	Antibodies used for flow cytometry were validated via relevant citations and inclusion of positive and negative controls. In all cases validation statements are also available on the manufacture's website. Other than flow cytometry, antibodies were not used in any other experiments to validate.

Animals and other organisms

Policy information about studies involving animals; ARRIVE guidelines recommended for reporting animal research

Laboratory animals	Mice of the following strains were used in this study: C57Bl/6J and B10.RIII background strains as well as genetically modified mice that lack the indicated genes and/or contain TCR-Transgene. All mice were bred individually in specific pathogen-free (SPF) conditions at the VA Portland Health Care System, where they were maintained at 21C on 12 h light-dark cycle (6am to 6pm) and given free access to food and water. Since we do not observe a sex-bias for males vs. females in the Nod2-associated phenotype both genders were used experimentally (in a matched manner) between the ages of 6-10 wks.

Wild animals	No wild animals were used in this study.
Field-collected samples	No field animals were used in this study.
Ethics oversight	Studies were carried out in accordance with the US Department of Health and Human Services Guide for the Care and Use of Laboratory Animals and were performed under institutional IACUC protocols approved at the VA Portland Health Care System.

Note that full information on the approval of the study protocol must also be provided in the manuscript.

Human research participants

Policy information about studies involving human research participants

Population characteristics	The two related patients with Blau syndrome (confirmed heterozygous CARD15c.1147G->A mutation, leading to pGlu383Lys mutation) included a mother (Blau-2, 48-year-old female) and son (Blau-1, 17-year-old male), who had onset of disease at ages 15 and 9, respectively. Blau-1 had been previously treated with methotrexate and was on adalimumab to control dermatitis and arthritis at the time of blood draw. Blau-2 had been on adalimumab and infliximab in the past and was on abatacept, methotrexate, prednisone (5-15 mg daily), and alendronate to control arthritis, uveitis, and nodular skin lesions with granulomatous inflammation (confirmed by biopsy) at the time of blood draw.
Recruitment	There was no self-selection bias. The two patients with Blau syndrome were selected based on their previously confirmed mutation in NOD2 (heterozygous CARD15c.1147G->A mutation, leading to pGlu383Lys mutation) and their symptoms--both of which defined them as having Blau syndrome. Patients were treated at University of Minnesota were they were enrolled in this research study with their treating physicians Drs. Vehe and Binstadt. Conceivably, given the stated criteria for participation including confirmed Blau Syndrome and that they were patients of Dr. Vehe or Binstadt this presents a potential selection bias. This selection bias could impact the results if the two donors do not reflect the general Blau community. The healthy controls were gender and age-matched accordingly.
Ethics oversight	Studies were carried out under protocols approved by the Institutional Review Boards of the University of Minnesota and Oregon Health & Science University.

Note that full information on the approval of the study protocol must also be provided in the manuscript.

Flow Cytometry

Plots

Confirm that:

- [x] The axis labels state the marker and fluorochrome used (e.g. CD4-FITC).
- [x] The axis scales are clearly visible. Include numbers along axes only for bottom left plot of group (a 'group' is an analysis of identical markers).
- [x] All plots are contour plots with outliers or pseudocolor plots.
- [x] A numerical value for number of cells or percentage (with statistics) is provided.

Methodology

Sample preparation	For mouse cells: Eyes were enucleated and the intraocular lens removed. Pineal glands were removed from the skull cap. Single-cell suspensions were prepared from eyes or pineal glands by collagenase-digestion (1 mg/ml Clostridium histolyticum Collagenase D, Roche) for 40 min at 37C and sequential filtration through a 70- and 40-µm strainer as described60. Single cell suspensions from spleens were prepared from immunized mice, and erythrocytes were lysed using red blood cell lysis buffer (Sigma)<br><br>For human samples: Human CD4+ T cell stimulations were carried out on viable frozen PBMCs from Blau syndrome patients vs. healthy, gender-matched controls. Peripheral blood was collected into BD Vacutainer ® CPT Mononuclear Cell Preparation tubes (Sodium Citrate) and PBMCs were isolated per manufacturer's instructions (BD Bioscience) within 24 h of collection. Cells were subsequently processes for flow cytometry.
Instrument	Cells were analyzed on an LSRFortessa (BD Biosciences).
Software	FlowJo (Becton, Dickinson, & Company, Franklin Lakes, NJ, USA) was used for analysis, allowing compensations for spectral overlaps within each sample.
Cell population abundance	Cells were stained with live/dead AQUA (ThermoFischer). Dead cells were excluded based on viability dye staining so that only live cell events were collected.
Gating strategy	Following fixation (4% paraformaldehyde), cells were analyzed on an LSRFortessa (BD Biosciences). Dead cells were excluded based on viability dye staining so that only live cell events were collected. FlowJo 10.0 (Becton, Dickinson, & Company, Franklin Lakes, NJ, USA) was used for analysis, allowing compensations for spectral overlaps within each sample. Gating used defined criteria based on control samples stained with corresponding isotype control antibodies (IC Ab) and equal number of total live events were collected. Live cells were gated from forward and side scatter plots in identical fashion for each group, after which frequencies and numbers of leukocyte subpopulations were determined from CD45+ gated cells.

NOTE: The gating strategies have been defined in detail in the Methods section along with defining markers for the identification of distinct cell populations.

☐ Tick this box to confirm that a figure exemplifying the gating strategy is provided in the Supplementary Information.
